# *Kcnn4*/KCa3.1 inhibition blunts polycystic kidney disease progression in mouse models

**DOI:** 10.1172/jci.insight.191311

**Published:** 2025-10-22

**Authors:** Guanhan Yao, Almira Kurbegovic, Camila Parrot, William Foley, William Roman, Seth L. Alper, Marie Trudel

**Affiliations:** 1 Molecular Genetics and Development, Institut de recherches cliniques de Montréal, Montréal, Québec, Canada.; 2 Division of Nephrology and Vascular Biology Research Center, Beth Israel Deaconess Medical Center, Boston, Massachusetts, USA.; 3 Department of Medicine, Harvard Medical School, Massachusetts, USA.; 4 Broad Institute of MIT and Harvard, Cambridge, Massachusetts, USA.; 5 Department of Medicine, Department of Biochemistry and Molecular Medicine, Faculty of Medicine, Université de Montréal, Montréal, Québec, Canada.

**Keywords:** Genetics, Nephrology, Drug therapy, Genetic diseases, Mouse models

## Abstract

The mechanisms underlying cyst growth and progression in Autosomal Dominant Polycystic Kidney Disease (ADPKD) remain unresolved. Since cyst expansion requires epithelial salt and water secretion likely involving basolateral membrane K^+^ recycling, we investigated the role of *KCNN4*-encoded K^+^ channel KCa3.1, inhibited by the potent, pharmacospecific, well-tolerated antagonist, senicapoc. We hypothesized that genetic and/or pharmacological inactivation of *KCNN4*/KCa3.1 would slow PKD progression. *KCNN4* was upregulated in kidneys of patients with ADPKD and of mechanistically distinct PKD mouse models. Cyst expansion in *Pkd1^–/–^* murine metanephroi was stimulated by KCa3.1 agonist and was prevented/reversed by senicapoc. In rapidly and/or slowly progressive mouse *Pkd1* models, *Kcnn4* inactivation slowed renal cyst growth; attenuated PKD-stimulated cAMP and ERK/Myc signaling pathways; reduced PKD-associated ciliary elongation, cell proliferation, and fibrosis; improved renal function; and prolonged survival. Importantly, senicapoc treatment of *Pkd1* mouse models phenocopied most effects of *Kcnn4* inactivation. This first study on the efficacy of KCa3.1 inhibition in PKD progression recommends senicapoc as a clinical trial candidate for ADPKD.

## Abstract 2

Genetic and pharmacological inhibition of *Kcnn4*/KCa3.1 prevents or regresses cysts *ex vivo*, delays polycystic kidney disease *in vivo* and suggests a clinical trial candidate.

## Introduction

Autosomal dominant polycystic kidney disease (ADPKD) affects ~1:1,000 individuals and frequently progresses to end-stage kidney disease. Approximately 85% of ADPKD is caused by mutations in the *PKD1* gene. ADPKD progression correlates with increases in kidney volume, resulting from slow enlargement of cysts arising in only ~3%–5% of the nephrons. The growing cysts eventually compress adjacent normal renal tubules, leading to death of noncystic nephrons.

The relentlessly increasing cyst volume that characterizes ADPKD is governed by epithelial cell secretion of lumenal fluid, accompanied by cell proliferation, interstitial fibrosis, alteration in ciliary structure and function, metabolic reprogramming, inflammation, and apoptosis. These cystic changes are paralleled by early dysregulation of signaling pathways and their downstream effectors and targets, including altered cAMP signaling, activation of MEK/ERK and multiple additional protein kinases, and altered transcriptional regulation through upregulation of c-Myc and other transcription factors.

Cyst transepithelial fluid secretion is mediated via polarized ion channels and transporters. All net ion transport contributing to cyst growth is accompanied by net water transport. Plasma vasopressin levels are elevated in patients with ADPKD, while urinary concentrating ability is decreased (1, 2). Vasopressin stimulation of adenylyl cyclase-coupled vasopressin V2 receptor (V2R) is the major source of renal tubular epithelial cell cAMP in ADPKD. V2R-stimulated cAMP levels contribute to increased transepithelial fluid secretion via coordinated protein kinase A–mediated (PKA-mediated) activation of apical water channel aquaporin 2 and apical Cl^–^ channel cystic fibrosis transmembrane conductance regulator (CFTR). Indeed, rare patients with ADPKD with cystic fibrosis mutations exhibit reduced rates of total kidney volume increase compared with family members without cystic fibrosis (3, 4). TMEM16A/ANO1 and other Cl^–^ channels likely contribute to fluid secretion (5). Mouse studies have suggested pharmacological inhibition of CFTR (6), CFTR corrector (7), or genetic inactivation of *Tmem16a* chloride channels (8) can slow renal cyst enlargement. Pharmacological targeting of salt and water secretion is, thus, a promising strategy to slow cyst growth (9).

Active Cl^–^ secretion by apical channels in cyst epithelial cells requires increased basolateral membrane Cl^–^ influx, principally via Na^+^-K^+^-2Cl^–^ cotransporter NKCC1/SLC12A2 (10). Maintenance of baseline and stimulated apical Cl^–^ secretion also requires basolateral uptake of Na^+^(via NKCC1 and Na^+^,K^+^-ATPase) and recycling of K^+^ (involving basolateral K^+^ channels). Prominent among the incompletely defined routes of basolateral K^+^ recycling in secretory epithelial cells and cultured ADPKD cyst epithelial cells is the *KCNN4*-encoded Ca^2+^-activated K^+^ channel of intermediate conductance, KCa3.1 (known in erythrocytes as the Gardos channel) (11–15). In some heterologous systems, KCa3.1 has also exhibited regulation by cAMP and PKA (16, 17). *KCNN4* gain-of-function mutations cause the autosomal dominant hemolytic anemia, hereditary xerocytosis (18–21), and KCa3.1 inhibition attenuates some indices of disease severity in murine (22, 23) and human sickle cell disease (24). *Kcnn4*-KO mice are grossly normal and reproduce normally (25, 26), with moderate hypertension reported in one model. *Kcnn4^–/–^* RBCs and T lymphocytes are unchanged in resting cell volume, but regulatory volume decrease is impaired (25, 27). *Kcnn4* in erythroid precursor cells is regulated by c-Myc (28), a major driver of cystogenesis in ADPKD epithelial cells and in PKD mouse models (29–31). KCa3.1 activation or inhibition in cultured human ADPKD cyst epithelial cells modulates CFTR-mediated Cl^–^ secretion without affecting cell proliferation (9, 32). This report suggests that KCa3.1 could be a therapeutic target for inhibition to slow disease progression. Whereas studies with Cl^–^ channel inhibitors slow or abolish cyst expansion in cell culture, these drugs are either unapproved for human use or are clinically dose limiting. However, the role of KCa3.1 in cyst initiation and growth remains to be investigated ex vivo or in animal models of renal cystic disease.

Several *Pkd1* mouse models exhibiting widely differing rates of bilateral renal cyst growth and disease progression can serve to study pathogenesis and test therapeutic approaches. Mice homozygous for *Pkd1-*null alleles initiate renal cysts from E15.5, but perinatal lethality complicates use for pharmacologic studies. Other early-onset, rapidly progressive renal *Pkd1* loss-of-function models expressing severe disease include *Pkd1^f/f^*; *KspCre (Pkd1*^cKO^*)* (33) and hypomorphic *Pkd1^V/V^* mice (34, 35). Two more slowly progressive *Pkd1* transgenic mouse models, *^SB^Pkd1* (SB regulator elements) and *Pkd1^wt^*/*Pkd1_TAG_*, express full-length mouse *Pkd1* with preferential renal epithelial (*^SB^Pkd1*) or systemic (*Pkd1^wt^*) overexpression (36, 37). Both models resemble human disease in their slow progression accompanied by increased renal expression of *Pkd1* mRNA and Polycystin-1 protein (29, 38–41), but they differ in genetic mechanism from the hypomorphic human ADPKD alleles. Both hypomorphic and overexpressing mouse models share with human ADPKD increased renal epithelial cell proliferation, apoptosis, inflammation, and fibrosis.Two approved ADPKD treatments can slow disease progression. V2R inhibitor tolvaptan (Jynarque) can delay need for renal replacement up to 8 years (42). Somatostatin receptor inhibitors pasireotide and octreotide can also slow disease progression (43). However, both treatment types can exhibit dose-limiting side effects, and occasional hepatotoxicity of tolvaptan further highlights the need for additional candidate ADPKD treatments.

We hypothesized, inspired by Albaqumi et al. (32), that *Kcnn4* genetic inactivation or pharmacological inhibition of KCa3.1 would effectively treat ADPKD, as KCa3.1 inhibitor senicapoc has shown both target engagement and tolerability in human trials. We identified KCa3.1/*Kcnn4* as a major contributor to cyst enlargement that is upregulated in human ADPKD kidneys and in kidneys of all examined *Pkd1* mouse models. Genetic and pharmacologic modulation in *Pkd1*-mutant mouse-derived metanephroi demonstrated that KCa3.1 upregulation induces and drives cyst formation and growth, whereas downregulation reduces cyst enlargement and even reverts previously formed cysts. *Kcnn4* genetic inactivation in 2 mechanistically orthologous *Pkd1* mouse models considerably slowed development of morphological and cell biological hallmarks of ADPKD. Administration of KCa3.1 inhibitor senicapoc attenuated cyst growth and other indices of disease progression in all 4 tested *Pkd1* mouse models of ADPKD.

## Results

### Upregulation of renal KCNN4 in human ADPKD and Pkd1 mouse models.

**Figure 1:**
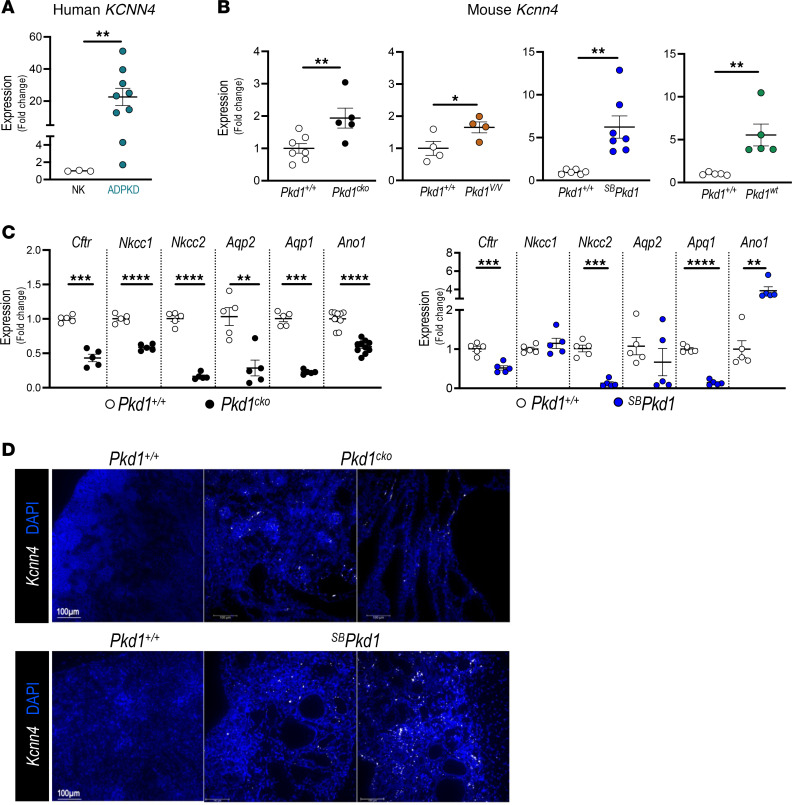
Selective upregulation of *KCNN4* in kidneys from human patients with ADPKD and from *Pkd1* mouse models. (**A**) Relative expression (qPCR) of human *KCNN4* in ADPKD (filled teal circles) and normal kidneys (NK, open circles). **P < 0.01, Student’s *t* test 1-tailed, Welch-corrected. (**B**) qPCR of mouse *Kcnn4* in kidneys from 4 *Pkd1* models and age-matched *Pkd1*^+/+^ controls (open circles): *Pkd1^cko^* (black, P10), *Pkd1^V/V^* (caramel, P10) *^SB^Pkd1* (blue, 2 mo), and *Pkd1^wt^* (green, 7 mo). *P < 0.05; **P < 0.01, Student’s *t* test, 1-tailed. (**C**) qPCR of mouse *Cftr*, *Nkcc1*, *Nkcc2*, *Aqp2*, *Aqp1*, and *Ano1* in P10 *Pkd1^cko^* kidneys (left, black) and 6–8 mo *^SB^Pkd1* kidneys (right, blue), versus age-matched *Pkd1*^+/+^ (open circles). **P < 0.01; ***P < 0.001; ****P < 0.0001, Student’s *t* test, 2-tail, Welch-corrected. (**D**) In situ hybridization of mouse kidney *Kcnn4* (RNAScope) from P5 *Pkd1^cko^,* 4 mo *^SB^Pkd1*, and age-matched *Pkd1*^+/+^, DAPI-counterstained. Scale bar: 100 μm.

To assess a possible role for *KCNN4* in human ADPKD progression, we first evaluated *KCNN4* expression by quantitative PCR (qPCR) and digital droplet PCR in human kidney tissue resected from patients with or without ADPKD. ADPKD kidneys consistently exhibited *KCNN4* RNA expression ~20-fold greater than did nominally normal kidneys (Figure 1A).

We next investigated *Kcnn4* expression in several *Pkd1* murine models of ADPKD. The Polycystin-1 dosage-reduced model *Pkd1^f/f^*; *KspCre* mice (*Pkd1*^cKO^) exhibits cysts at P2, with rapid cyst growth leading to death by ~P17 (31). The hypomorphic *Pkd1^V/V^* mouse model develops cysts at P4 and dies at day 25.0 ± 3.3 with enlarged cystic kidneys (35). Renal expression of *Kcnn4* in P10 *Pkd1^cko^* and *Pkd1^V/V^* mice was upregulated 2-fold compared with WT (Figure 1B). We also evaluated renal *Kcnn4* RNA expression in 2 transgenic ADPKD mouse models with slower cyst expansion and disease progression, *^SB^Pkd1* mouse (37) and *Pkd1^wt^* (36) (Figure 1B). *Kcnn4* RNA levels were ~5- to 6-fold higher than in WT counterparts. Similar renal *Kcnn4* upregulation characterized the nonorthologous SBM PKD mouse model (30) (Supplemental Figure 1A; supplemental material available online with this article; https://doi.org/10.1172/jci.insight.191311DS1). In contrast, *Kcnn4* RNA abundance was unchanged in liver, brain, heart, and spleen of *Pkd1^V/V^* and *^SB^Pkd1* mice and in *Pkd1^V/V^* pancreas (Supplemental Figure 1, B and C). Renal upregulation of *KCNN4* RNA is, thus, a shared feature of human ADPKD and of mouse ADPKD models with reduced or increased *Pkd1* gene dosage.

We assessed specificity of *Kcnn4* upregulation by comparing renal expression changes of additional selected ion transporters/channels. Renal RNA levels of the *Cftr*, *Nkcc1*, *Nkcc2*, *Aqp2,* *Aqp1*, and *Ano1* genes were all reduced in abundance in P10 *Pkd1^cKO^* versus WT mice, consistent with previous reports (44–46). Expression of these RNAs (except for ANO1*)* was reduced or unchanged versus WT in 4–6 months (mo) *^SB^Pkd1* kidneys (Figure 1C). Renal upregulation of *Kcnn4* RNA is thus both reproducible and specific across multiple mouse models of ADPKD, supporting potential role(s) in cyst enlargement. To assess *Kcnn4* spatial expression in cystic kidneys, we examined early- and adult-onset *Pkd1* mouse models of ADPKD by RNAScope (Figure 1D). P5 and 4 mo WT *Pkd1^+/+^* kidneys exhibited little or no *Kcnn4* signal in cortical, medullary, or papillary regions. P5 *Pkd1^cKO^* kidney sections revealed *Kcnn4* RNA signal in noncystic tubules, with increased *Kcnn4* signal in some cyst-surrounding cells and in interstitial regions (Figure 1D). *^SB^Pkd1* kidneys (4 mo) exhibited increased *Kcnn4* RNA signal versus WT, in cyst epithelial and surrounding cells (Figure 1D) with similar results in 6–7 mo *Pkd1^wt^* kidneys (not shown). Renal *Kcnn4* upregulation in all tested *Pkd1* mouse models of ADPKD suggests that *Kcnn*4 plausibly contributes to renal cyst growth in ADPKD.

### Kcnn4 regulates cystogenesis in mouse metanephroi.

Mouse WT *Pkd1*^+/+^ metanephroi cultured in vitro from E14.5 undergo cAMP-stimulated ureteric bud branching, tubule formation, and growth, without apparent morphological anomalies. *Kcnn4* is expressed at E14.5 and E16.5 in developing *Pkd1*^+/+^ or *Pkd1*^–/–^ kidneys (Supplemental Figure 1D), validating cultured metanephroi as an ex vivo experimental system in which to investigate KCa3.1 roles in cyst formation and enlargement. This pattern of embryonic *Kcnn4* expression, together with its very low levels in adult kidney, reinforces the concept that cystogenesis in ADPKD may represent a failed developmental switch to the adult renal molecular program.

**Figure 2:**
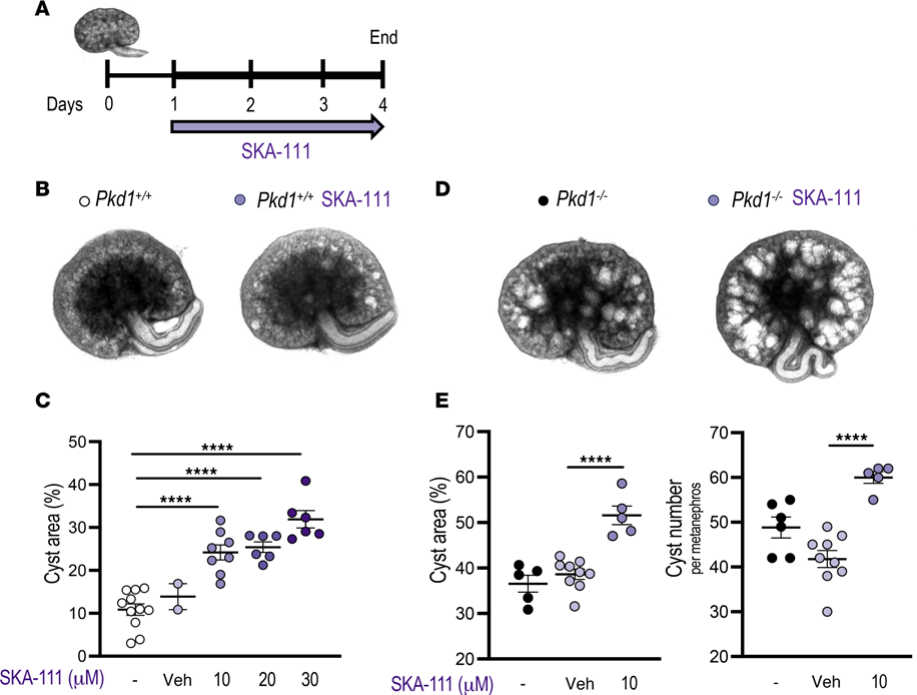
KCa3.1 activator SKA-111 promotes cystogenesis and/or cyst growth in metanephroi. (**A**) E14.5 (day 0) *Pkd1^+/+^* and *Pkd1^–/–^* metanephroi were stimulated with 100 µM cAMP (thicker line) and treated with vehicle (veh) or KCa3.1 activator SKA-111 (arrow, days 1–4). (**B**) Representative *Pkd1^+/+^* metanephroi after 4 days without or with SKA-111. (**C**) Cyst area (%) of *Pkd1^+/+^* metanephroi without (open) or with vehicle (gray) or SKA-111 (violet, concentration-dependent intensity). ****P < 0.0001, ANOVA. (**D**) Representative *Pkd1^–/–^* metanephroi after 4 days without or with 10 µM SKA-111. (**E**) Cyst area (%) and number of *Pkd1^–/–^* metanephroi without (black) or with vehicle (gray) or SKA-111 (violet). ****P < 0.0001, ANOVA.

cAMP exposure of E14.5 *Pkd1*^+/+^ metanephroi elicits rare tubular dilatations and occasional small cysts, whereas cAMP-treated *Pkd1*^–/–^ metanephroi develop multiple large cysts over 4–6 days in culture (Figure 2) (47). We therefore assessed whether the KCa3.1 agonist SKA-111 modulates cAMP-stimulated cystogenesis or cyst enlargement in *Pkd1*^+/+^ or *Pkd1*^–/–^ metanephroi (Figure 2A). Treatment of *Pkd1*^+/+^ metanephroi with increasing SKA-111 concentrations led to dose-dependent cystogenesis and > 2-fold increased percentage of cyst area (cystic index) (Figure 2, B and C). SKA-111 treatment of *Pkd1*^–/–^ metanephroi, already prone to larger cyst development, further increased cyst area ~34% and cyst number ~44% versus vehicle (Figure 2, D and E). Thus, KCa3.1 activity has the potential to modulate ex vivo cyst development and progression.

**Figure 3:**
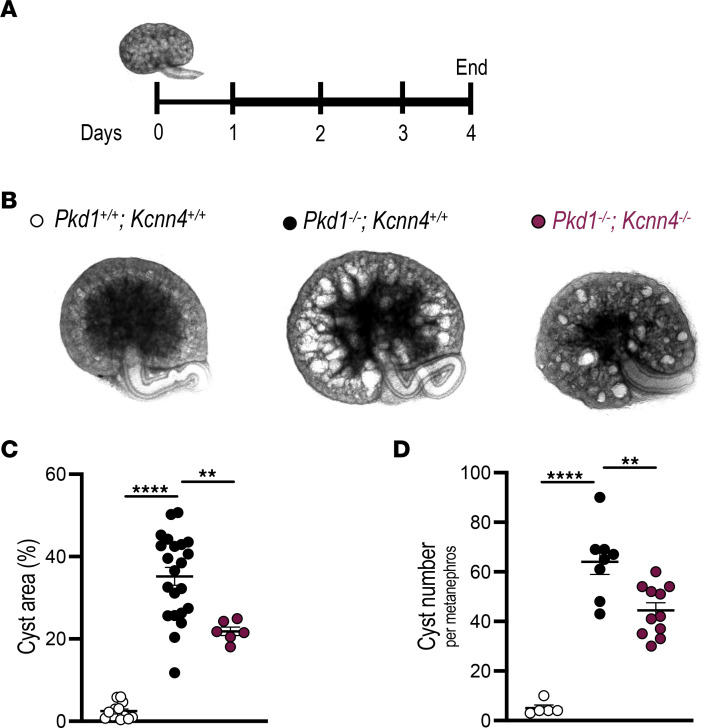
Genetic inactivation of *Kcnn4* attenuates cyst development in mouse metanephroi. (**A**) Metanephroi were cAMP-stimulated (100 µM, days 1-4, thicker line). (**B**) Representative day 4 metanephroi: *Pkd1^+/+^*(open circles), *Pkd1^–/–^* (black), and *Pkd1^–/–^*;*Kcnn4^–/–^* (burgundy). (**C**) Cyst area (%) in metanephroi color-coded per **B**. **P < 0.01; ****P < 0.0001, ANOVA. (**D**) Cyst number color-coded per **B**. **P < 0.01; ****P < 0.0001, ANOVA.

Although *Kcnn4*^–/–^ mice were reported grossly normal (25), kidney histology was not examined. Absence of microscopic renal anomalies and apparent fibrosis in *Kcnn4^–/–^* adult mouse kidneys (Supplemental Figure 2) confirmed utility of testing effects of *Kcnn4* inactivation on renal cyst appearance and enlargement. We therefore intercrossed *Pkd1^+/–^*;*Kcnn4^–/–^* mice to assess cyst formation in metanephroi (Figure 3A) from *Pkd1*^–/–^;*Kcnn4^–/–^* embryos and compared with metanephroi from *Pkd1*^–/–^;*Kcnn4^+/+^* and *Pkd1*^+/+^;*Kcnn4^+/+^* controls obtained from intercrosses of *Pkd1^+/–^*;*Kcnn4^+/+^* mice. *Pkd1*^–/–^;*Kcnn4^–/–^* metanephroi (Figure 3B) exhibited ~40% lower cyst area (Figure 3C) and ~30% lower cyst number (Figure 3D) than in *Pkd1*^–/–^;*Kcnn4^+/+^* metanephroi, suggesting that KCa3.1 expression contributes not only to cyst enlargement but perhaps also to cystogenesis.

**Figure 4:**
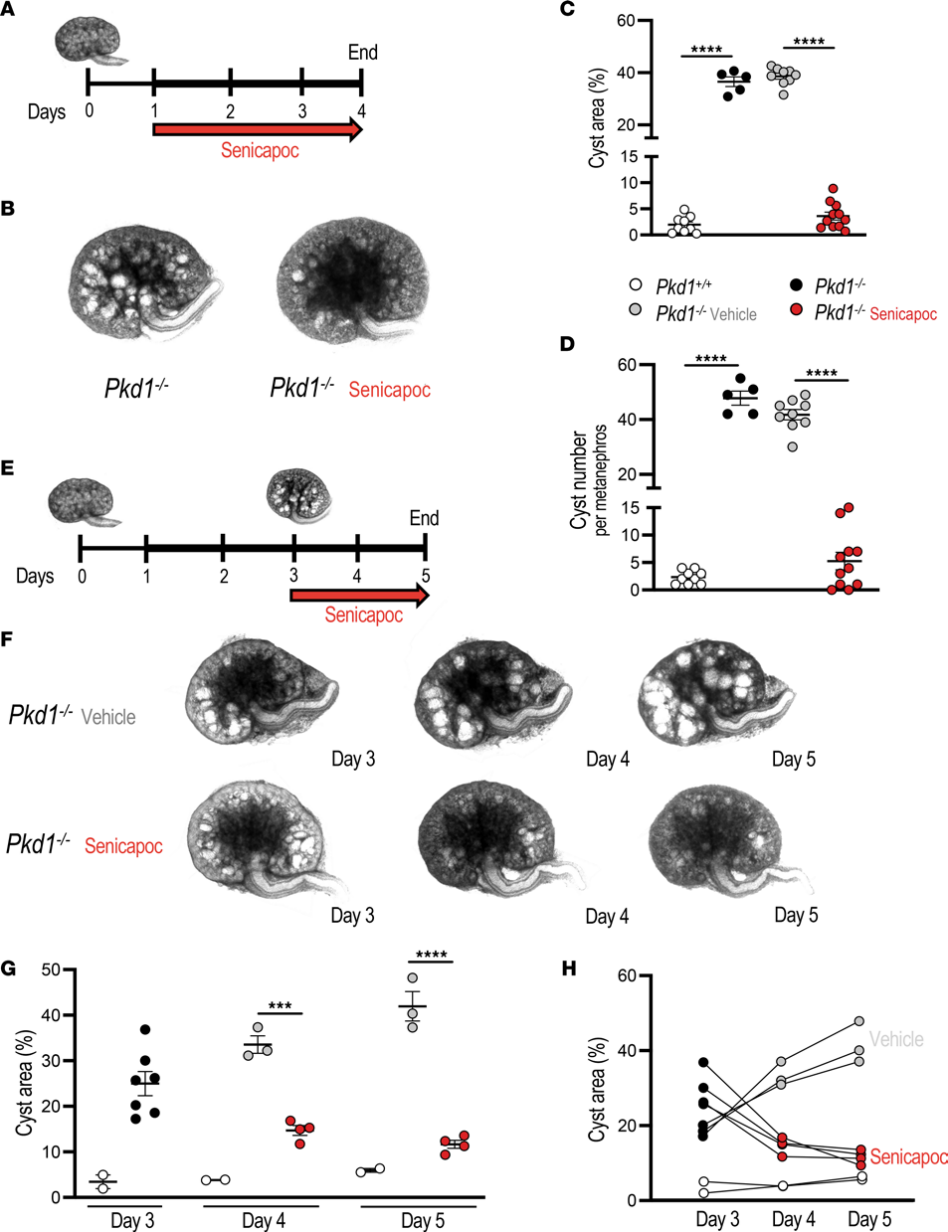
KCa3.1 antagonist senicapoc inhibits ex vivo cyst growth and promotes regression of preformed cysts. (**A**) cAMP-stimulated metanephroi (100 µM, thicker line) with vehicle or senicapoc (days 1–4, red arrow). (**B**) Representative day 4 *Pkd1^–/–^* metanephroi treated with vehicle or senicapoc (20 µM). (**C**) Cyst area (%) in day 4 metanephroi: *Pkd1**^+/+^*** (open circles), *Pkd1^–/–^* (black), treated with vehicle (gray), or senicapoc (20 µM, red). ****P < 0.0001, ANOVA. (**D**) Cyst number in day 4 metanephroi color-coded per **C**. ****P < 0.0001, ANOVA. (**E**) Cyst reversal experiment (**F**–**H**): *Pkd1^–/–^* metanephroi cAMP-stimulated days 1–5 (thicker line) were treated days 3–5 with vehicle or 20 µM senicapoc (red arrow). (**F**) Time series of *Pkd1^–/–^* metanephroi treated with vehicle or 20 µM senicapoc from days 3–5. (**G**) Cyst area progression in day 3 *Pkd1**^+/+^*** and *Pkd1**^–/–^*** metanephroi treated days 3–5 with vehicle (gray) or senicapoc (red). ***P < 0.001; ****P < 0.0001, ANOVA. (**H**) Cyst area progression time course of individual *Pkd1* metanephroi treated with vehicle or senicapoc.

We then questioned whether the well-tolerated KCa3.1 inhibitor senicapoc (24, 48, 49) could slow cyst growth in cultured metanephroi (Figure 4A), similarly to *Pkd1* genetic inactivation. *Pkd1*^–/–^ metanephroi cultured in the presence of 20 µM senicapoc (Figure 4B) exhibited ~90% reduction in both cyst area and number. The structurally related KCa3.1 inhibitor TRAM-34, effective in cultured cells (32, 50), also reduced cyst area of *Pkd1*^–/–^ metanephroi by ~25%–30% at 20 and 40 µM (Figure 4, C and D, and Supplemental Figure 3). Senicapoc at these concentrations exhibits antiinflammatory and antiinnate immune activities as well as channel inhibition. The similar responses of *Pkd1^–/–^* metanephroi to both senicapoc and TRAM-34 support important contribution(s) of KCa3.1 function to cyst enlargement in metanephroi.

To more closely model treatment of patients with ADPKD, we evaluated senicapoc efficacy in established cysts. *Pkd1*^–/–^ metanephroi underwent cyst growth for 72 hours (h) before 48h further supplementation with senicapoc or vehicle (Figure 4E). Whereas vehicle-treated cystic metanephroi displayed progressively increased cyst area and number, delayed senicapoc treatment was associated with marked regression of cyst area and number to near WT levels (Figure 4, F–H). These results provide further evidence of KCa3.1-dependent cyst growth activity in *Pkd1*^–/–^ metanephroi.

### Potentiation of senicapoc-mediated inhibition of ex vivo cyst growth by Cl^–^ channel inhibitors.

To gain insight into KCa3.1 interactions and mechanism in renal epithelial fluid secretion during cyst enlargement, we next evaluated cyst enlargement in *Pkd1*^–/–^ metanephroi treated with senicapoc alone or in combination with inhibitors of apical Cl^–^ channels TMEM16A/Ano1 and CFTR, previously shown to block cyst growth (51, 52). Senicapoc (5–20 µM) dose-dependently reduced cyst area in *Pkd1*^–/–^ metanephroi (Supplemental Figure 4A). TMEM16a inhibitor CaCCinh-A01 (A01,10–30 µM) only minimally reduced cyst area in *Pkd1*^–/–^ metanephroi. However, in the additional presence of 5 µM senicapoc (itself minimally inhibitory), 30 µM A01 substantially reduced cyst area, suggesting a synergistic effect (Supplemental Figure 4B). Treatment of *Pkd1*^–/–^ metanephroi with 2–20 µM CFTR inhibitor PPQ102 progressively decreased cystic surface area. Senicapoc (5 µM) addition to these *Pkd1*^–/–^ metanephroi potentiated inhibition of cyst enlargement (Supplemental Figure 4C), as with A01. Thus, metanephroi responses to these combined treatments highlight possible interactions of KCa3.1 and Cl^–^ channels in transepithelial secretory ion transport driving cyst enlargement.

### Upregulated signaling pathways associated with cyst enlargement in 4 Pkd1 mouse models.

**Figure 5:**
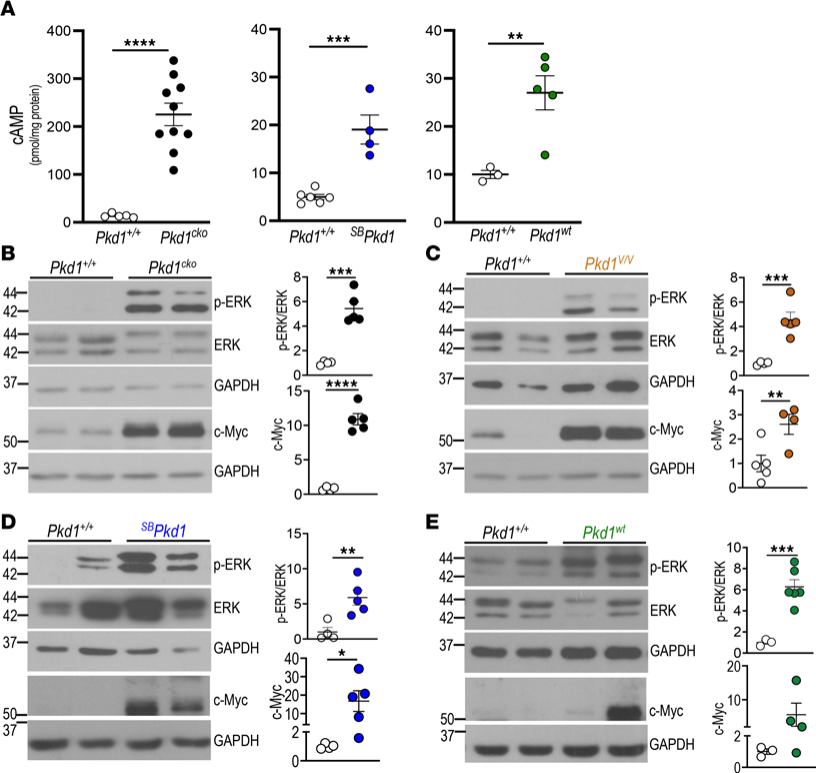
Signaling effectors associated with cyst enlargement in kidneys of 4 *Pkd1* models. (**A**) Renal cAMP in P10 WT *Pkd1**^+/+^*** (open circles) and *Pkd1^cko^* mice (black); 5–6 wk *Pkd1**^+/+^*** and *^SB^Pkd1* mice (blue); 6–8 mo *Pkd1**^+/+^*** and *Pkd1^wt^* mice (green). **P < 0.01; ***P < 0.001; ****P < 0.0001. (**B**) Immunoblots of pERK/ERK and c-Myc (left) and densitometric quantitation (right) in P10 *Pkd1**^+/+^*** (open circles) and *Pkd1^cko^* kidneys (black). ***P < 0.001; ****P < 0.0001. (**C**) Immunoblots of pERK/ERK and c-Myc in P10 *Pkd1**^+/+^*** (open circles) and *Pkd1^V/V^* kidneys (caramel), quantitated as in **B**. **P < 0.01; ***P < 0.001. (**D**) Immunoblots of pERK/ERK and c-Myc in 5–6 wk *Pkd1**^+/+^*** (open circles) and *^SB^Pkd1* kidneys (blue) quantitated per **B**. *P < 0.05; **P < 0.01. (**E**) Immunoblots of pERK/ERK and c-Myc in 6–8 mo *Pkd1**^+/+^*** (open circles) and *Pkd1^wt^* kidneys (green) quantitated per **B**. ***P < 0.001, **A**–**E**, Student’s *t* test, 1-tailed.

*Kcnn4* upregulation in 4 orthologous mouse models of ADPKD, together with KCa3.1’s role in ex vivo cyst enlargement, prompted our systematic assessment of several major effectors of the fluid secretion pathways driving cyst growth. Elevated renal cAMP levels are associated with ADPKD progression, and cAMP has been reported in some conditions to activate KCa3.1 (17). We therefore measured cAMP levels in kidney extracts from mouse ADPKD models with different rates of disease progression. Compared with age-matched controls, renal cAMP levels were elevated up to 25-fold in extracts from the rapidly progressive *Pkd1^cKO^* model (Figure 5A). In extracts from more slowly progressive *^SB^Pkd1* and *Pkd1^wt^* models, cAMP was also elevated ~4- and ~2.6-fold. The nonorthologous SBM mouse model also showed markedly increased cAMP levels (Supplemental Figure 5). Thus, renal cAMP content was increased in multiple ADPKD mouse models of differing rates of progression.

Since ERK and c-Myc are both downstream mediators of cAMP signaling and contribute to PKD-associated cell proliferation (53, 54), we next examined ERK and c-Myc activation states in ADPKD mouse models. *Pkd1^cKO^* and *Pkd1^V/V^* (P10) kidneys with rapidly progressive disease showed 4- to 5-fold greater increases in phospho-ERK (pERK)/total ERK compared with control *Pkd1*^+/+^ kidneys and showed up to 10-fold higher levels of c-Myc (Figure 5, B and C, and Supplemental Figure 6). Similarly, kidneys from *^SB^Pkd1* and *Pkd1^wt^* mice with moderately progressive disease exhibited 5- to 10-fold increased pERK/ERK ratio and increased c-Myc expression versus age- and strain-matched *Pkd1*^+/+^ kidneys (Figure 5, D and E, and Supplemental Figure 6), consistent with our previous demonstration of c-MYC upregulation in human ADPKD (29). The above data emphasize the similarity of these regulatory responses in all ADPKD mouse models tested.

### Genetic inactivation of Kcnn4 attenuates severity of cystic disease in an early-onset Pkd1 mouse model.

**Figure 6:**
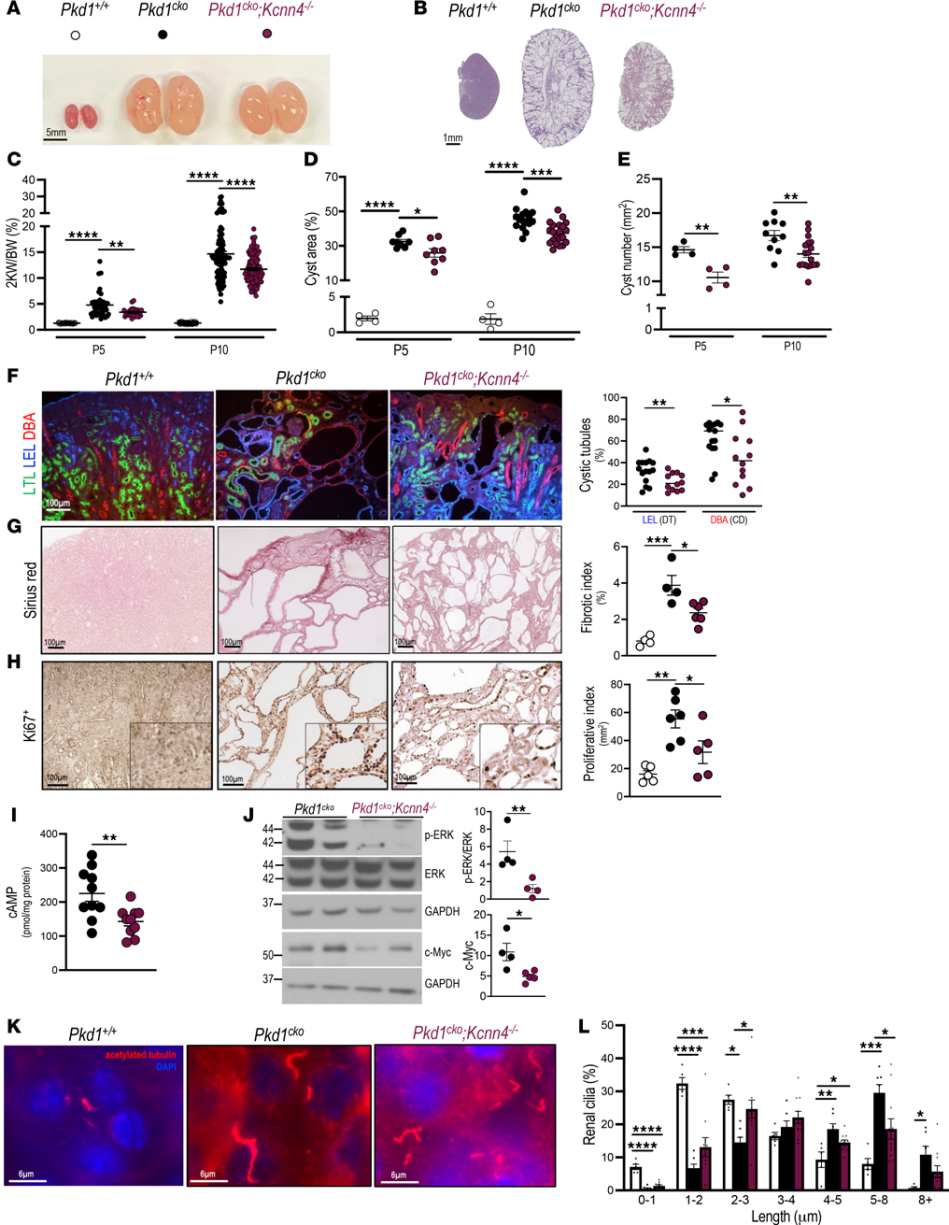
*Kcnn4* genetic inactivation attenuates PKD pathology in *Pkd1^cko^* mice. (**A**) Representative P10 kidneys from *Pkd1**^+/+^*** (open circles), *Pkd1^cko^* (black), and *Pkd1^cko^*;*Kcnn4^–/–^* mice (burgundy). Scale bar: 5 mm. (**B**) Representative H&E stained axial sections of P10 *Pkd1**^+/+^***, *Pkd1^cko^*, and *Pkd1^cko^*;*Kcnn4^–/–^* kidneys. Scale bar: 1 mm. (**C**) 2KW/BW (%) in P5 and P10 mice, color-coded as in **A**, *Pkd1**^+/+^*** (P5, *n* = 39; P10, *n* = 117), *Pkd1^cko^* (P5, *n* = 38; P10, *n* = 98), and *Pkd1^cko^*;*Kcnn4^–/^^–^* (P5, *n* = 26; P10, *n* = 110). **P < 0.01; ****P < 0.0001, ANOVA. (**D**) Kidney cyst area color-coded as in **A**. *P < 0.05; ***P < 0.001; ****P < 0.0001, ANOVA. (**E**) Kidney cyst number color-coded as in **A**. **P < 0.01, Student’s *t* test, 1-tailed. (**F**) Merged images of cystic tubules in P5 *Pkd1**^+/+^***, *Pkd1^cko^*, and *Pkd1^cko^*;*Kcnn4^–/–^* kidneys stained with nephron segment–specific lectin markers for proximal (LTL), distal (LEL), and collecting duct (DBA) (Supplemental Figure 7A for unmerged images). Graph shows percent lectin-positive cystic tubules, genotypes color-coded as in **A**. *P < 0.05; **P < 0.01, Student’s *t* test, 1-tailed. Proximal tubule dilatation was subthreshold for cyst designation. (**G**) Sirius red staining of renal fibrosis in P10 *Pkd1**^+/+^***, *Pkd1^cko^* and *Pkd1^cko^*;*Kcnn4^–/–^* mice. Graph shows percent fibrotic index, genotypes color-coded as in **A**. *P < 0.05; ***P < 0.001, ANOVA. (**H**) Epithelial cell proliferation (Ki67^+^) in P10 *Pkd1**^+/+^***, *Pkd1^cko^*, and *Pkd1^cko^*;*Kcnn4^–/–^* kidneys. Graph shows Ki67^+^ cells/mm^2^, genotypes color-coded as in **A**. *P < 0.05; **P < 0.01, ANOVA. (**I**) Kidney cAMP in P10 *Pkd1^cko^*; *Kcnn4^–/–^* (burgundy circles) versus *Pkd1^cko^* mice (black, data from Figure 5A). **P < 0.01, Student’s *t* test, 1-tailed. (**J**) pERK/ERK and c-Myc immunoblots of P10 *Pkd1^cko^* and *Pkd1^cko^*;*Kcnn4^–/–^* kidneys, GAPDH loading control. Graph of normalized pERK/ERK and c-Myc levels, genotypes color-coded as in **A**. *P < 0.05; **P < 0.01, Student’s *t* test, 1-tailed. (**K**) α-Acetylated tubulin in cilia of P10 *Pkd1**^+/+^***, *Pkd1^cko^* and *Pkd1^cko^*;*Kcnn4^–/–^* kidneys. (**L**) Binned ciliary lengths in P10 *Pkd1**^+/+^***, *Pkd1^cko^*, and *Pkd1^cko^*;*Kcnn4^–/–^* kidney sections, color-coded as in **A**. *P < 0.05; **P < 0.01; ***P < 0.001; ****P < 0.0001, ANOVA. Scale bars: 6 µm.

Delayed cyst growth in *Pkd1*^–/–^ metanephroi genetically deficient in *Kcnn4* spurred in vivo studies of KCa3.1 function in mouse ADPKD models by generating *Pkd1^cko^* mice with inactivated *Kcnn4* alleles. *Pkd1^cko^*;*Kcnn4^–/–^* (P10) kidneys were smaller than those of *Pkd1^cko^*, with greater parenchymal preservation (Figure 6, A and B), consistent with decreased P5 and P10 respective kidney/body weight ratios (2KW/BW) of ~30% and ~20%, versus *Pkd1^cko^* (Figure 6C). Semiquantitative histologic analysis of *Pkd1^cko^*;*Kcnn4^–/–^* kidneys revealed 20% reduction in cyst area and number versus *Pkd1^cko^* (Figure 6, D and E).

*Pkd1^cko^* kidneys developed cysts and tubular ectasia in distal tubules and collecting ducts (Figure 6F), whereas proximal cysts were absent or infrequent (not shown), as detected by costaining with nephron segment markers, *Lotus tetragonolobus* lectin (LTL; proximal tubules), Lycopersicon esculentum lectin (LEL; distal convoluted tubules), and *Dolichos biflorus* agglutinin (DBA; collecting duct) (Figure 6F and Supplemental Figure 7A). Genetic inactivation of *Kcnn4* in *Pkd1^cko^* kidneys did not alter this segmental pattern, but it did reduce by ~30% the number of cysts of distal tubule or collecting duct origin (Figure 6F). Despite attenuation of the cystic phenotype, *Pkd1^cko^*;*Kcnn4^–/–^* lifespan was not prolonged, nor was blood urea nitrogen (BUN) significantly lower, compared with *Pkd1^cko^* mice. Nonetheless, the up to ~5-fold increased fibrosis (Sirius red) in P10 *Pkd1^cko^* kidneys was significantly reduced by *Kcnn4* inactivation (Figure 6G). Proliferative index (Ki67 positivity) in *Pkd1^cko^* kidney tubular epithelial and interstitial cells was also strongly attenuated in *Pkd1^cko^*;*Kcnn4^–/–^* kidneys (Figure 6H). These results support *Kcnn*4’s important contributions to both PKD renal fibrosis and proliferation.

PKD-associated upregulation of cAMP levels in *Pkd1^cko^* kidneys was strikingly decreased by *Kcnn4* inactivation (Figure 6I). Molecular signaling responses of elevated ERK and c-Myc in *Pkd1^cko^* kidneys were also markedly diminished in the absence of *Kcnn4* expression (Figure 6J), suggesting KCa3.1 involvement in the cAMP proliferative pathway. As increased cAMP levels elongate primary cilia in IMCD cells (55), we noted that cilia of P10 *Pkd1^cko^* tubular epithelial cells were consistently longer than in *Pkd1*^+/+^ kidneys (Figure 6, K and L). Notably, *Pkd1^cko^*;*Kcnn4^–/–^* renal tubular cells exhibited a significant leftward shift in ciliary length distribution towards] the WT pattern (Figure 6L), consistent with reduction of mean ciliary length (3.76 ± 0.57 µm; *P* < 0.005) versus P10 *Pkd1^cko^* (5.08 ± 0.67 µm). These results support a role of *Kcnn4*-encoded KCa3.1 in cAMP signaling, cyst enlargement, cyst cell proliferation, and ciliary length determination in rapidly progressive mouse models of ADPKD.

### Genetic inactivation of Kcnn4 markedly improves kidney disease in an adult Pkd1 mouse model.

**Figure 7:**
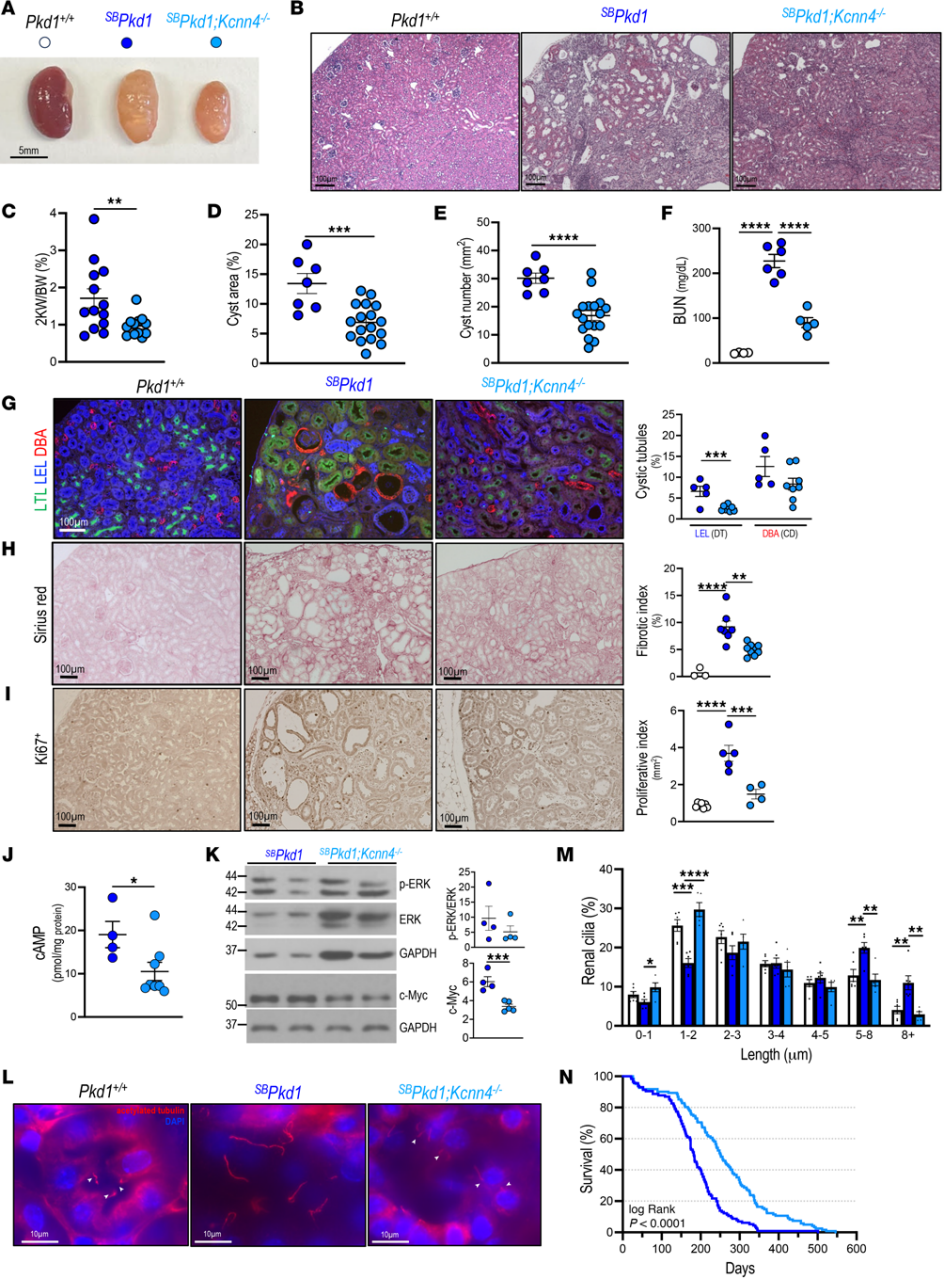
*Kcnn4* inactivation in *^SB^Pkd1* mice attenuates cellular and molecular indices of disease progression, kidney function decline, and extends lifespan. (**A**) Kidneys from 6–8 mo *Pkd1**^+/+^***(open circles), *^SB^Pkd1* (blue), and *^SB^Pkd1*;*Kcnn4^–/–^* mice (light blue). Scale bar: 5 mM. (**B**) Histologic sections (H&E) of *Pkd1**^+/+^***, *^SB^Pkd1* and *^SB^Pkd1*;*Kcnn4^–/–^* kidneys. Scale bar: 100 µM. (**C**) 2KW/BW ratio in 6 mo mice, genotypes color-coded as in **A**. **P < 0.01, Student’s *t* test, 1-tailed. (**D**) Cyst area in *Pkd1**^+/+^***, *^SB^Pkd1*, and *^SB^Pkd1*;*Kcnn4^–/–^* kidney sections, color-coded as in **A**. ***P < 0.001, Student’s *t* test, 1-tailed. Scale bar: 100 µM. (**E**) Cyst number/mm^2^ in *Pkd1**^+/+^***, *^SB^Pkd1*, and *^SB^Pkd1*;*Kcnn4^–/–^* kidneys color-coded as in **A**. ****P < 0.0001, Student’s *t* test, 1-tailed. (**F**) Blood urea nitrogen (BUN) in 6 mo *Pkd1**^+/+^***, *^SB^Pkd1*, and *^SB^Pkd1*;*Kcnn4^–/–^* mice color-coded as in **A**. ****P < 0.0001, ANOVA. (**G**) Merged images (unmerged in Supplemental Figure 7B) of cystic tubules from 2 mo *Pkd1**^+/+^***, *^SB^Pkd1* and *^SB^Pkd1*;*Kcnn4^–/–^* mice stained by segment-specific lectin markers LTL, LEL, and DBA. Graph shows percent lectin-positive cystic tubules, genotypes color-coded as in **A**. ***P < 0.001, Student’s *t* test, 1-tailed. Proximal tubule dilatation was below detection threshold. (**H**) Sirius red staining of fibrosis in 6–8 mo *Pkd1**^+/+^***, *^SB^Pkd1* and *^SB^Pkd1*;*Kcnn4^–/–^* kidneys. Scale bar: 100 µM. Graph shows percent fibrotic index, genotypes color-coded as in **A**. **P < 0.01; ****P < 0.0001, ANOVA. (**I**) Ki67 immunostaining of 6–8 mo *Pkd1**^+/+^***, *^SB^Pkd1* and *^SB^Pkd1*;*Kcnn4^–/–^* kidneys. Graph shows Ki67^+^ cells/mm^2^, genotypes color-coded as in **A**. ***P < 0.001; ****P < 0.0001, ANOVA. Scale bar: 100 µM. (**J**) Kidney cAMP in 2 mo *^SB^Pkd1*;*Kcnn4^–/–^* versus *^SB^Pkd1* mice (data from Figure 5A), genotypes color-coded as in **A**. *P < 0.05, Student’s *t* test, 1-tailed. (**K**) pERK/ERK ratio and c-Myc immunoblots of 6 mo *^SB^Pkd1* and *^SB^Pkd1*;*Kcnn4^–/–^* kidneys. Graph of normalized pERK/ERK ratio and c-Myc levels, genotypes color-coded as in **A**; ***P < 0.001, Student’s *t* test, 1-tailed. (**L**) α-Acetylated tubulin in cilia (arrowheads) of *Pkd1**^+/+^***, *^SB^Pkd1* and *^SB^Pkd1*;*Kcnn4^–/–^* kidneys. Scale bar: 20 µM. (**M**) Binned ciliary lengths in *Pkd1**^+/+^***, *^SB^Pkd1* and *^SB^Pkd1*;*Kcnn4^–/–^* kidney sections, color-coded as in **A**. *P < 0.05; **P < 0.01; ***P < 0.001; ****P < 0.0001, ANOVA. (**N**) Kaplan-Meier survival curves of *^SB^Pkd1* (blue, *n* = 115) and *^SB^Pkd1*;*Kcnn4^–/–^* mice (light blue, *n* = 121). *P* < 0.0001, Log-rank test.

To investigate a potential role of KCa3.1 in the more slowly progressive mouse ADPKD model *^SB^Pkd1*, we generated *^SB^Pkd1* mice with genetic inactivation of *Kcnn4*. *^SB^Pkd1*;*Kcnn4^–/–^* mouse kidneys were smaller (Figure 7A), with fewer cysts (Figure 7B) and ~44% lower 2KW/BW ratio than *^SB^Pkd1* kidneys (Figure 7C). Cyst area and cyst number were both lower in *^SB^Pkd1*;*Kcnn4^–/–^* kidneys than in *^SB^Pkd1* kidneys (Figure 7, D and E). Increased BUN in *^SB^Pkd1* was ~60% reduced in the absence of *Kcnn4* expression (Figure 7F). Genetic inactivation of *Kcnn4* decreased the proportions of cystic distal tubules and collecting ducts (Figure 7G and Supplemental Figure 7B). Interstitial fibrosis evident in aging *^SB^Pkd1* kidneys was reduced ~50% in the absence of *Kcnn4* (Figure 7H). Elevated renal cell proliferation in *^SB^Pkd1* mice, as evidenced by Ki67 staining, was nearly normalized in *^SB^Pkd1* mice lacking *Kcnn4* expression (Figure 7I). Pathologically increased renal cAMP content, pERK/ERK ratio, and c-Myc abundance in *^SB^Pkd1* kidneys were reduced in the absence of *Kcnn4* expression (Figure 7, J and K). Typically elongated primary cilia of *^SB^Pkd1* kidneys were normalized by genetic ablation of *Kcnn4*, as measured by ciliary length distribution in renal epithelial cells (Figure 7, L and M). Most dramatically, *Kcnn4* genetic inactivation in *^SB^Pkd1* mice extended median lifespan by ~40% and maximal lifespan by 58% (Figure 7N). As *^SB^Pkd1* mice display no extrarenal pathology (37), their delayed demise was plausibly attributable to slowed onset of renal failure. These data demonstrate a crucial role for *Kcnn4* in regulation of multiple cellular indices of PKD progression in the slowly progressive *^SB^Pkd1* mouse model of ADPKD, including cyst enlargement, cell proliferation, renal fibrosis, and ciliary length control. The beneficial effects of *Kcnn4* genetic inhibition in orthologous *Pkd1* mouse models, therefore, prompted tests of KCa3.1 pharmacologic inhibition on disease progression.

### KCa3.1 inhibitor senicapoc attenuates the course of PKD progression in 4 mouse models.

**Figure 8:**
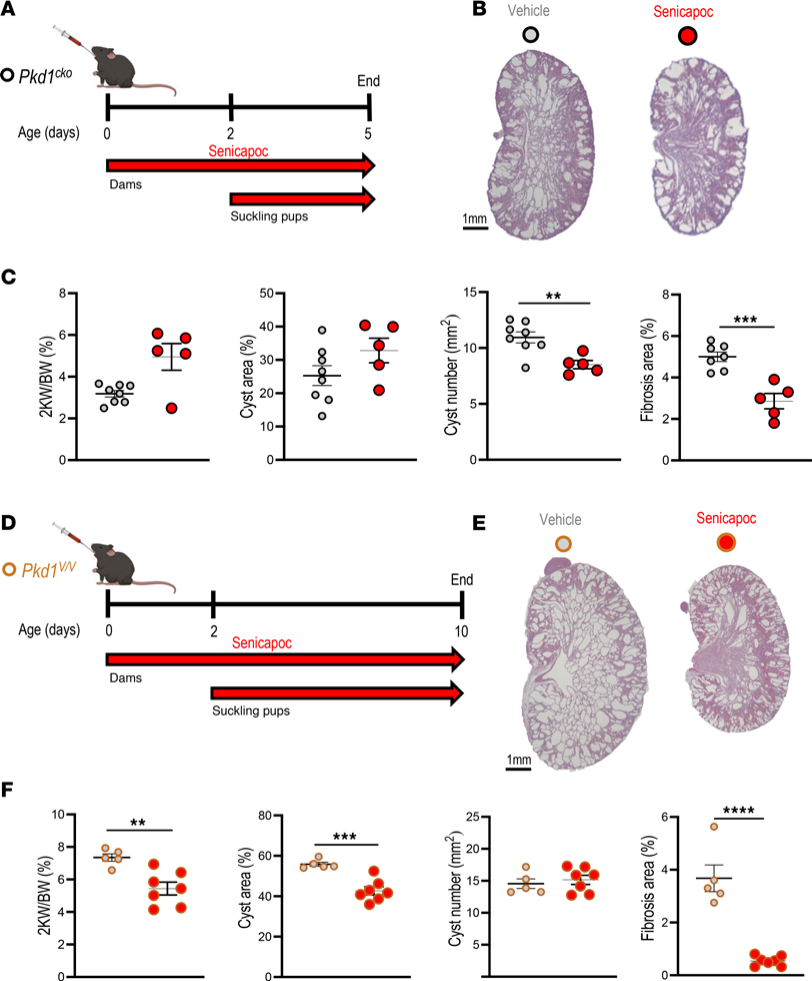
KCa3.1 inhibitor senicapoc reduces cyst growth in 2 early-onset *Pkd1* mouse models. (**A**) Oral treatment of *Pkd1^cko^* mice (black-rimmed circle) with 120 mg/kg senicapoc (red arrow). Senicapoc or vehicle was administered daily by oral gavage to suckling mothers from P0 to P5 and, in addition, directly into mouths of suckling pups from P2 to P5. (**B**) H&E kidney sections from P5 *Pkd1^cko^* mice treated with vehicle (PEG/Cremophor; black-rimmed gray circle) or senicapoc (120 mg/kg; black-rimmed red circle). Scale bar: 1 mm. (**C**) 2KW/BW ratios, percent cyst area, cyst number, and fibrotic index in vehicle- or senicapoc-treated *Pkd1^cko^* pups, color-coded per **B**. **P < 0.01; ***P < 0.001, Student’s *t* test, 1-tailed. (**D**) Oral treatment of *Pkd1^V/V^* mice (caramel-rimmed circle) with 120 mg/kg senicapoc (red arrow). Senicapoc or vehicle was administered daily by oral gavage to suckling mothers from P0 to P10 and, in addition, directly into mouths of suckling pups from P2 to P10. (**E**) H&E kidney sections of P10 *Pkd1^V/V^* mice treated with vehicle (caramel-rimmed gray circle) or senicapoc (caramel-rimmed red circle). Scale bar: 1 mm. (**F**) The 2KW/BW ratios, percent cyst area, cyst number, and fibrotic index in vehicle- or senicapoc-treated *Pkd1^V/V^* pups, color-coded per **E**. **P < 0.01; ***P < 0.001; ****P < 0.0001, Student’s *t* test, 1-tailed.

We tested senicapoc efficacy in early-onset, rapidly progressive loss-of-function *Pkd1^cko^* and *Pkd1^V/V^* mice and in slowly or moderately progressive *^SB^Pkd1* and *Pkd1^wt^* transgenic PKD models with gradual disease progression more closely resembling human *PKD1*-mutant ADPKD. Initiation times and durations of senicapoc treatment were chosen according to phenotype severity and to minimize attrition in vehicle-treated groups. Senicapoc (120 mg/kg/day) or vehicle (PEG/cremophor) was delivered orally to pups of both sexes from P2 to P5 for *Pkd1^cko^* (Figure 8A) or to P10 for *Pkd1^V/V^* (Figure 8D), concomitant with gavage of suckling dams from birth to P5 or P10, respectively. Mouse pups maintained normal growth without apparent adverse effects. Senicapoc treatment decreased cyst number ~20%–25% in P5 *Pkd1^cko^* mice without parallel decrease in cyst area or 2KW/BW ratio but reduced renal fibrosis ~50% (Figure 8, B and C). Longer senicapoc treatment of P10 *Pkd1^V/V^* mice reduced 2KW/BW ratio and cyst area ~25%–30% and reduced fibrosis ~85% versus vehicle-treated *Pkd1^V/V^* mice (Figure 8, E and F).

**Figure 9:**
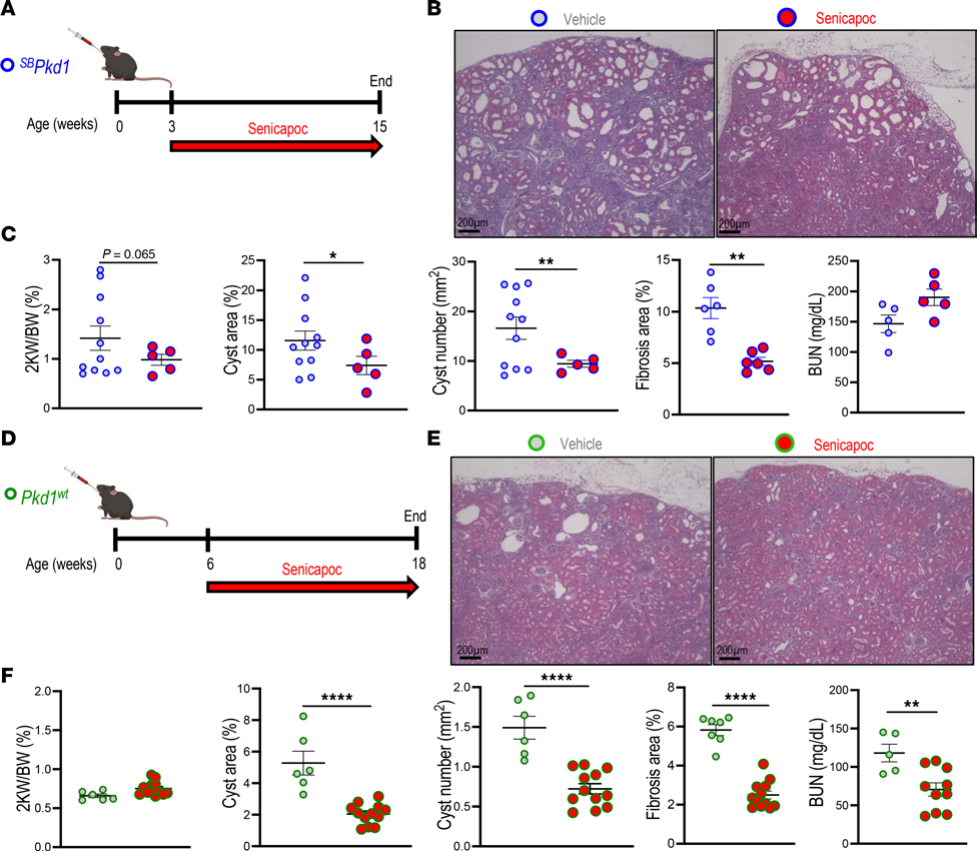
KCa3.1 inhibitor senicapoc reduces cyst growth in 2 late-onset *Pkd1* mouse models. (**A**) Treatment of postweaning (3 wk) *^SB^Pkd1* mice (blue-rimmed circle) with 120 mg/kg senicapoc (red arrow, 12 wk daily oral gavage). (**B**) H&E kidney sections from 15 wk *^SB^Pkd1* mice treated 12 wk with vehicle (blue-rimmed gray circle) or senicapoc (120 mg/kg, blue-rimmed red circle). Scale bar: 200 μm. (**C**) The 2KW/BW ratio, percent cyst area and number, fibrotic index, and BUN in vehicle- or senicapoc-treated *^SB^Pkd1* mice color-coded per **B**. *P < 0.05; **P < 0.01, Student’s *t* test, 1-tailed, Welch-corrected. (**D**) Treatment of 6 wk *Pkd1^wt^* mice (green-rimmed circle) with 120 mg/kg senicapoc (red arrow, 12 wk daily oral gavage). (**E**) H&E kidney sections from 18 wk *Pkd1^wt^* mice treated 12 wk with vehicle (green-rimmed gray circle) or senicapoc (120 mg/kg, green-rimmed red circle). Scale bar: 200 μm. (**F**) The 2KW/BW ratio, percent cyst area and number, fibrotic index, and BUN in vehicle- or senicapoc-treated *Pkd1^wt^* mice, color-coded per **E**. **P < 0.01; ****P < 0.0001, Student’s *t* test, 1-tailed.

*^SB^Pkd1* mice (from age 3 weeks [wk]) and *Pkd1^wt^* mice (from age 6 wk) were treated 12 wk by daily gavage with 120 mg/kg senicapoc or vehicle (PEG/cremophor) (Figure 9, A and D). The different ages of treatment initiation reflect the times at which detectable cysts appear in the 2 models. Growth curves of mice treated 12 wk with drug or vehicle were very similar (Supplemental Figure 8). BUN was unchanged in senicapoc-treated *^SB^Pkd1* mice, but 2KW/BW ratio, cyst area, and cyst number decreased (or trended so), with ~50% reduced fibrosis (Figure 9, B and C). Whereas senicapoc treatment did not decrease 2KW/BW ratio in *Pkd1^wt^* mice, cyst area and numbers were reduced 40%–60%, with renal fibrosis reduced 50%–60% (Figure 9, E and F). Notably, BUN was reduced 40% in *Pkd1^wt^* mice treated 12 wk with 120 mg/kg/day senicapoc, consistent with slowed renal functional decline (Figure 9F). These results document that pharmacological inhibition of KCa3.1 partially phenocopies genetic inactivation of *Kcnn4* in slowing PKD progression in mouse models. The data provide the first in vivo evidence to our knowledge supporting therapeutic potential of senicapoc as a well-tolerated oral treatment to slow cyst growth and slow total kidney volume increase in patients with ADPKD.

## Discussion

This study has identified *Kcnn4*-encoded Ca^2+–^activated potassium channel KCa3.1 as a major regulator of PKD pathogenesis ex vivo and in vivo. Upregulation of *KCNN4* expression in human ADPKD kidneys, as well as in kidneys of several mechanistically distinct *Pkd1* mutant mouse models, is consistent with a role of KCa3.1 in cystic kidney disease. Ex vivo exposure of metanephroi to a pharmacologic activator of KCa3.1 potentiated cyst initiation and progression, whereas exposure to pharmacologic inhibitors of KCa3.1 either prevented cyst enlargement or promoted complete cyst regression. Genetic ablation of *Kcnn4* in multiple orthologous murine *Pkd1* models attenuated or normalized several upregulated PKD cellular and signaling pathways and, in some models, improved renal function and prolonged lifespan. Treatment with KCa3.1 inhibitor senicapoc completely or partially phenocopied genetic deletion of *Kcnn4* in 4 *Pkd1* mouse models, 2 featuring early disease onset with rapid progression and 2 of later onset with slower rates of progression. These results position senicapoc, a drug well-tolerated in human clinical trials across multiple disease settings, as a promising candidate for Phase 1b–2a clinical trial in human ADPKD.

Striking upregulation of *KCNN4* expression in kidneys of all tested patients with ADPKD was recapitulated in all mechanistically distinct *Pkd1* animal models examined, whether associated with loss of *Pkd1* expression, partial *Pkd1* loss of function, or *Pkd1* overexpression. These results validate significant increases of *KCNN4* expression in ADPKD kidney cells heterozygous for *PKD1* mutant Q2556X (56) and in ADPKD single nucleus RNA-seq (snRNA-Seq) data from humans with advanced disease (57). Increased renal *Kcnn4* RNA expression in cyst epithelium and surrounding tissue was consistent with contributory roles in cystogenesis and/or cyst enlargement. The association was further supported by the selectively increased *Kcnn4* expression relative to several other tested ion channels and transporters contributing to cyst fluid secretion. *Kcnn4/KCNN4* expression is near or below sc/snRNA-Seq detection thresholds in adult kidneys or isolated nephron segments of mouse (58–62), rat (63), zebrafish (64), and human (https://esbi.nhlbi.nih.gov/MRECA/Nephron/), whereas *Kcnn4/KCNN4* expression was easily detected in embryonic kidneys of mouse (Supplemental Figure 1D) (65), zebrafish (64), and human (66). Elevated renal expression pattern of *KCNN4* in ADPKD kidneys is consistent with our longstanding view that cystic epithelium reflects either failure to mature from a renal developmental program (67) or pathological dedifferentiation toward an immature epithelial phenotype (68, 69). The latter includes transition from an absorptive to (“less differentiated”) secretory phenotype (70, 71). Importantly, our data suggest *Kcnn4* as a procystogenic regulator in mouse and human ADPKD kidneys.

Our studies in *Pkd1* mouse models demonstrate strong correlation of *Kcnn4* expression status with cAMP/ERK/cMyc signaling in progression of cyst growth. Kidney cyst expansion in mice, whether in settings of decreased or increased *Pkd1* expression, was consistently associated with elevated levels of cAMP, known to promote growth, proliferation, and secretion in cultured human ADPKD epithelial cells (47, 72, 73). cAMP may also indirectly activate KCa3.1 activity in some experimental systems (32, 74, 75). Increased in vivo cAMP levels accompanied by activation of ERK signaling in our mouse models reinforce a form of signaling crosstalk previously reported in ADPKD cells (76, 77). The downstream effector of ERK, c-Myc (78, 79), is markedly increased in kidneys of our mouse models. Moreover, transgenic *Myc* overexpression in mouse kidneys induces a PKD phenotype (30), with *Kcnn4* upregulation and increased cAMP levels. As a central proliferative and metabolic node, c-Myc is viewed a master regulator in ADPKD (31, 80). Concomitant increases of renal *Myc* and *Kcnn4* expression in our PKD mouse models corresponding with those in human ADPKD (29) are consistent with *c-Myc* regulation of *Kcnn4* expression (28). The similar endogenous developmental expression profiles of *Myc* and *Kcnn4* in human and mouse further supports a regulatory interaction. Conversely, reductions in cAMP, ERK, and c-Myc levels, along with decreased cyst growth upon *Kcnn4* inactivation in PKD mouse models, revealed a molecular signature dynamically linked to cyst formation. These results suggest a possible mechanistic relationship integrating cAMP, ERK, c-Myc, and *Kcnn4*/KCa3.1.

*Kcnn4*/KCa3.1 was procystogenic and prosecretory in mouse embryonic kidneys ex vivo by several criteria. The KCa3.1 agonist induced cyst formation and growth in WT mouse metanephroi in a concentration-dependent manner. KCa3.1 agonist also potentiated increased cyst formation in *Pkd1^–/–^* metanephroi. Genetic inactivation of *Kcnn4* in *Pkd1^–/–^* metanephroi markedly delayed cyst initiation and growth. This inactivation was phenocopied by exposure to KCa3.1 inhibitor, senicapoc, which nearly completely prevented cyst formation and growth in *Pkd1^–/–^* metanephroi. Delayed senicapoc treatment of already cystic *Pkd1^–/–^* metanephroi caused rapid cyst regression and restoration of a nearly normal tubular morphology in metanephroi. This mechanism during developmental stages may reflect underlying cell plasticity, as defined by cellular ability to change phenotype or identity in response to external stimuli, without alteration of genomic DNA sequence (81, 82). The result suggests possible small molecule–regulated plasticity manifest as reversal of the cystic tubular epithelium functional state, perhaps through activation of cellular regeneration and/or renal repair processes. The results indicate that KCa3.1 exerts not only procystogenic and prosecretory functions in mouse metanephroi but that it also might influence renal epithelial plasticity.

Genetic inactivation of *Kcnn4* in rapidly and moderately progressive *Pkd1* mouse models significantly delayed cyst growth, with substantial reduction in cellular, molecular, and physiologic hallmarks of PKD. Attenuated cyst growth in each disease model is demonstrated by reduced 2KW/BW ratio, cyst area, and cyst number. The important attenuation of renal fibrosis in these models is consistent with the antifibrotic actions of KCa3.1 inhibitors in mouse models of unilateral ureteral obstruction (83), diabetic kidney disease (84), and cisplatin-induced acute kidney injury (85), as well as in liver (86), lung (87, 88), and other extrarenal tissues. The marked reduction in renal cell proliferation by genetic inactivation of *Kcnn4* in tested PKD mouse models is consistent with antiproliferative effects of KCa3.1 inhibitors in T cells and vascular smooth muscle cells (89–91) but at variance with KCNN4 knockdown in epithelial cells or primary cultures of human ADPKD kidney cells (32). Our consistent findings of elevated renal cAMP content in tested *Pkd1* mouse models reinforces original observations of increased cAMP in cultured human cyst epithelial cells (92) and in the *Pkd1^RC/RC^* mouse model of ADPKD (93). Reduced cell proliferation in response to *Kcnn4* inactivation was associated with reduction in elevated levels of cAMP, ERK phosphorylation, and Myc abundance in murine PKD kidneys. Ciliary elongation detected in *Pkd1* mouse models (36) was reversed upon *Kcnn4* genetic inactivation, consistent with reduction in cAMP levels. Of particular interest, genetic inactivation of *Kcnn4* in moderately progressive disease models not only attenuated deterioration of renal function but also extended life span. These striking outcomes paralleled cellular and physiologic improvements, with partial normalization of PKD-associated alterations in signaling.

Pharmacological inhibition of KCa3.1 by oral administration of senicapoc phenocopied, to a large degree, the beneficial effects of *Kcnn4* KO in PKD mouse models, without apparent toxicity. Administration of senicapoc at seemingly high dose was justified by the short 1h to 2.5h half-life of senicapoc in murine plasma (49) versus the long ~12.8 day plasma half-life in humans (94). The estimated human equivalent dose suggests clinical translatability of our mouse dosing to below the 10-40 mg/day doses tested without toxicity in humans. Our results in 2 rapidly progressive *Pkd1* mouse models strongly support senicapoc’s ability to delay early-stage increases in renal cyst number and fibrosis with only 5 days’ treatment and its ability to slow cyst enlargement and fibrosis over a 10-day treatment course. Considering the rapid progression of cyst growth, short treatment duration and very short drug half-life combined with only twice-a-day administration, the potential therapeutic effect of senicapoc was very likely underestimated. Once-daily oral senicapoc treatment of the 2 more slowly progressive *Pkd1* mouse models for 12 wk markedly attenuated renal cyst growth and number, reduced fibrosis, and slowed deterioration of renal function. Changes in disease indices (2KW/BW ratio, cystic index, BUN, fibrosis) in response to senicapoc treatment indicated efficacy at least equivalent to that reported for tolvaptan in polycystic *Pkd1*^RC/RC^, iKspCre-*Pkd1*^del^, *pcy* mice, and *PCK* rats (95–98). Our findings thus demonstrate that *Kcnn4*-encoded KCa3.1 is a regulator of PKD progression with substantial therapeutic potential.

Although genetic inactivation of *Kcnn4* and treatment with the KCa3.1 inhibitor senicapoc both slowed cyst growth, neither intervention eliminated cyst growth. Activity of additional cyst epithelial cell K^+^ channels may contribute to basolateral K^+^ recycling required to sustain ongoing apical secretion of Cl^–^ and water into the cyst lumen. Most transcripts appear decreased or unchanged in ADPKD kidneys (57), consistent with our data and with previous observations in mouse (44, 46, 96) and humans (45, 99). However, comparative transcriptome analysis of human “normal” and ADPKD renal epithelial cell lines revealed upregulation of voltage-dependent K^+^ channel KCNQ1 and of the KCNS1 silent regulator of voltage-dependent KCNB K^+^ channels (56). Relationships between KCa3.1-driven ADPKD cyst formation and enlargement and other possibly contributing K^+^ channels remain to be determined.

Beneficial outcomes of senicapoc treatment in all tested *Pkd1* mouse models strongly recommend trial of senicapoc as a therapeutic approach to ADPKD treatment. Senicapoc is well tolerated in humans, even in those affected with sickle cell disease (24, 48, 100), asthma (Pfizer), and Alzheimer disease (49). Senicapoc has not been associated with hepatotoxicity in humans or mice. Although we have shown here senicapoc’s therapeutic potential as a single agent, senicapoc may also prove useful in reducing dosage of tolvaptan, the only currently approved ADPKD treatment. We therefore suggest that combination therapy of senicapoc and reduced-dose tolvaptan might increase patient compliance by reducing tolvaptan polyuria, polydipsia, and hepatotoxicity. Such combination therapy could also preserve or enhance, additively or synergistically, tolvaptan-associated effects on reducing growth in total kidney volume and slowing renal function decline. Our ex vivo results also suggest that senicapoc could potentiate or synergize with selective inhibitors of cyst epithelial Cl^–^ channels, when available.

Our study has several limitations. First, mouse models of ADPKD only partially recapitulate human ADPKD. Second, senicapoc trials in mice require at least twice-a-day dosing to maintain plasma drug levels adequate for target engagement, posing safety challenges for gavage or i.p. administration over periods of weeks to months. We conducted our long-term experiment on slowly progressive model adults with once-a-day dosing. We were nonetheless able to show partial senicapoc efficacy in all 4 genetically distinct PKD mouse models tested. The longer half-life of senicapoc in humans allows reliable achievement of drug levels consistent with target engagement. Third, further genetic experiments restricting *Kcnn4* KO to epithelial cells, as opposed to fibroblasts, myeloid, lymphoid, and other cells, are needed to delineate each cell type’s contribution to PKD progression in mouse models.

In conclusion, our results demonstrate that both genetic inactivation and pharmacological inhibition of *Kcnn4*/KCa3.1 can decrease and delay progression of PKD ex vivo and in multiple *Pkd1* mouse models. Beneficial outcomes of senicapoc treatment reinforce the therapeutic strategy of targeting epithelial fluid secretion system to halt ADPKD progression. Our study on *Kcnn4*/KCa3.1 inhibition recommends advancement of senicapoc pharmacotherapy into clinical trial in patients with ADPKD, initially as sole agent and subsequently, as part of combination therapy.

## Methods

### Sex as a biological variable.

As ADPKD affects males and females equally in the models studied. We report data from pooled males and females in roughly equal numbers.

### Metanephroi culture.

E14.5 embryonic kidneys dissected from *Pkd1^+/–^* mice intercrosses were PCR-genotyped to select *Pkd1^+/+^* and *Pkd1^–/–^* metanephroi and were then placed on transparent 0.4 mm inserts (Falcon) in 12 or 24 well plates (Fisher). Basal compartment culture medium was DMEM:F12 plus sodium bicarbonate, HEPES, Insulin-Transferrin-Selenium cocktail, prostaglandin E2, and penicillin and streptomycin (Wisent). Metanephroi were cultured < 6 days in a humidified 37°C incubator. Reagents added to media 24h after dissection with replacement every 48h included, 8-Br-cAMP (Sigma), SKA-111 (Aobious), senicapoc (BOC Sciences) or TRAM-34 (Tocris), PPQ102 (Tocris) and CaCCinh-A01 (Millipore-Sigma), and vehicle (0.1% DMSO, 3-fold below detectable effect on metanephroi). Metanephroi were brightfield-imaged daily at 4X (Axiovert S100TV). Cyst counts and areas were measured (Volocity, Quorum Technologies).

### Animal models and genotyping.

*Pkd1^–/–^* (*Pkd1^tm1Som^*) (101), *Pkd1^f/f^* (*Pkd1^tm2Ggg^*) (102), *Ksp-cadherin*;*Cre* (Tg[Cdh16-cre]91Igr) (103), *Kcnn4^–/–^* (Kcnn4^tm1Jemn^) (25) and knock-in *Pkd1^V/V^* (*Pkd1*^tm1.1Fqi^) mice (34, 35) were gifts of S. Somlo (Yale, New Haven, CT), G. Germino (NIDDK, Bethesda, MD), P. Igarashi (Stony Brook Univ, NY), J. Melvin (formerly NIDDS, Bethesda, MD), and F. Qian (U Maryland, Baltimore, MD). Transgenic lines overexpressing full-length *Pkd1* gene, *^SB^Pkd1* (line 39, Tg[*Pkd1*]39Mtru) and *Pkd1_TAG_* 26 (*Pkd1^wt^*, Tg[*Pkd1*]26MTru) were previously described (36, 37). All mouse lines were individually bred into C57BL/6J background. These mice were crossed with *Kcnn4^–/–^* mice to obtain *Pkd1^–/–^*;*Kcnn4^–/–^* metanephroi, and *Pkd1^cko^*;*Kcnn4^–/–^* and *^SB^Pkd1*;*Kcnn4^–/–^* mice. Genotypes were identified by PCR as previously (31, 104). Supplemental Table 1 lists PCR primers.

### Mouse tissue samples.

Kidneys and blood were collected at P5 and P10 for *Pkd1^cko^* and *Pkd1^V/V^*, and at 1–2 mo and 6–8 mo for *^SB^Pkd1*, *Pkd1^wt^* and corresponding age-matched WT mice. Kidney weights and body weights were recorded. Each kidney was hemisectioned, with 1 half snap-frozen for cAMP measurement and RNA/protein extraction, and 1 half formalin-fixed overnight for paraffin-embedding, histochemistry, in situ hybridization and Ki67 staining. P10 *Pkd1^cko^* and 6–8 mo *^SB^Pkd1* mice with/without *Kcnn4* inactivation were perfused and kidneys OCT-embedded for α-acetylated tubulin immunostaining.

### RNA isolation and qPCR analysis.

Surgically excised human ADPKD and nominally normal kidneys (29) and mouse renal and extrarenal tissues were extracted for RNA by TRIzol/chloroform and reverse transcribed. Duplicate qPCR reactions carried out (TaqMan master mix) and predesigned duplex gene expression assays for human *KCNN4* (4453320, FAM-MGB) were normalized to control (*HPRT,* HS00158470-m1, VIC-MGB) (Applied Biosystems). Triplicate qPCR reactions for *Kcnn4*, *Aqp1*, *Aqp2*, *Nkcc1*, *Nkcc2*, *Cftr,* and *Ano1* were normalized to S16 and/or β-actin. Supplemental Table 2 lists qPCR primers. Data from QuantStudio Real-Time PCR Software were analyzed in Excel/Prism and expressed as fold change.

### Protein extraction and immunoblotting.

Frozen kidneys were homogenized in RIPA buffer (20 mM Tris [pH 7.5], 2 mM EDTA pH 8, 150mM NaCl and 0.5% Triton X100) supplemented with PMSF (1 mM, Sigma) and proteinase inhibitor cocktail buffer (Sigma). Protein concentration (BCA or Bradford assay) was standardized versus bovine serum albumin (BSA, Sigma). Equal amounts of total protein denatured 10 min at 95°C in Laemmli buffer were separated in 10% or 12% polyacrylamide gels by SDS-PAGE and transferred overnight at 4°C to PVDF or nitrocellulose membrane, blocked 6 h at 4°C in PBST with 0.1% Tween-20 and 5% milk, and then incubated overnight with primary anti-c-Myc, anti-pERK, anti-ERK, or anti-GAPDH antibodies; rinsed multiple times; and incubated 4h at 4°C with secondary antibodies. Supplemental Table 3 lists all antibodies, suppliers and catalogue numbers. Immunoblots were ECL-developed (Amersham) and exposed to X-ray film, with analysis by ImageJ or ImageLab.

### Histology and IHC.

Formalin-fixed kidney sections (4–5 µM) were stained with H&E, Sirius Red (0.1%; Sigma) or conjugated lectins (Supplemental Table 3). As previous studies revealed ~95% of WT tubule diameters < 22μm, with only 4.8% of tubule diameters ≥ 22μm, 22μm was selected as the threshold for “cystic tubules” or “cysts” (80). Cyst number, percentage of cyst area, percentage of fibrosis, and percentage of cystic tubules were analyzed by Northern Eclipse (Empix Imaging), QuPath (105), or Volocity.

### Proliferation assay.

Proliferation was detected by sequential incubation of kidney sections with anti-Ki67 antibody and diaminobenzidine visualization (DAB, Vector Laboratories) (Supplemental Table 3) analyzed by Northern Eclipse.

### RNAScope in situ hybridization.

Permeabilized kidney sections from P5 *Pkd1^cko^* and 4 mo *^SB^Pkd1* mice and age-matched controls were hybridized with mouse *Kcnn4* antisense probe (catalog 569381) and multiplex fluorescent reagent Kit v.2 (ACD). Slides were incubated with Opa1690 (Akoya Biosciences) for *Kcnn4*, then mounted in Prolong Gold (Invitrogen). ACD-negative controls served as background. Slides were imaged by DM6 microscope (Leica) as described (104). 

### Primary cilia measurement.

Perfused kidney cryosections were stained with anti-acetylated-α-tubulin antibody and anti-mouse IgG (Supplemental Table 3). Nuclei were DAPI stained. Sections were imaged by DM6 microscope. Primary cilia length was measured in Volocity by manual tracing, binning by length, and calculating percentage of total ciliated cells per bin.

### cAMP measurement.

Kidney lysate cAMP was measured by direct cAMP assay kit (Enzo) per manufacturer’s instructions. Frozen kidneys ground in liquid nitrogen were homogenized in 10 vol 0.1M HCl, before being centrifuged (600*g*). Supernatants were added to the assay plate, along with serially diluted cAMP standards. All samples were treated sequentially with cAMP antibody, substrate solution, and stop solution before being incubated 1h at 20^o^C. Absorption at 405 nm allowed calculation of cAMP content in pmol/mg protein.

### BUN measurement.

BUN was measured in mouse plasma samples using QuantiChrom Urea Assay Kit (BioAssay Systems DIUR-100) per manufacturer’s instructions.

### In vivo drug administration.

Each adult mouse model (3 wk *^SB^Pkd1*, 6 wk *Pkd1^wt^*, and 6 wk C57BL6/J) was separated into 3 groups: untreated, PEG/cremophor-treated (vehicle-treated) control and 120 mg/kg senicapoc in PEG/cremophor. Mice treated daily by oral gavage were sacrificed after 3 mo. Mothers of early onset models *Pkd1^cko^* and *Pkd1^V/V^* were treated with 120 mg/kg senicapoc in PEG/cremophor daily from birth by oral gavage to transmit through maternal milk. Pups were also orally dosed 120 mg/kg senicapoc in PEG/cremophor twice daily from P2 until P5 or P10. The 2KW/BW ratio, kidney cyst area and number, BUN and renal fibrosis were analyzed as above.

### Statistics.

Values were expressed as mean ± SD. Statistical analysis of data sets (GraphPad Prism) was by unpaired Student’s *t* test with and without Welch correction, ANOVA, or log-rank test as appropriate. *P* < 0.05 was considered statistically significant.

### Study approval.

All animal experiments were approved by the Institutional Animal Care Committee of Institut de recherches cliniques de Montréal and the Canadian Council of Animal Care (no. 2021-07). Human kidney specimens were from Vivette D’Agati (Pathology Department, Columbia University, New York, New York) under approved clinical investigation protocol.

### Data availability.

Primary values for all data points in graphs are reported and available in Supporting Data Values file.

## Author contributions

GY conducted and analyzed experiments, drafted some figures, and edited the final manuscript. AK developed methodology, conducted and analyzed experiments, interpreted data, drafted and edited the manuscript, and drafted and edited final figures. CP developed methodology, conducted and analyzed experiments, and edited the final manuscript. WF conducted and analyzed experiments and statistics, produced final figures, and edited final manuscript. WR conducted experiments. SLA conceived and contributed to work design, conducted experiments and interpreted data, and wrote and edited the manuscript and figures. MT conceptualized and designed the project, conducted and analyzed experiments, interpreted data, supervised the study, and wrote and edited the manuscript and figures. All coauthors read and approved the final manuscript.

## Funding support

This work is the result of NIH funding, in whole or in part, and is subject to the NIH Public Access Policy. Through acceptance of this federal funding, the NIH has been given a right to make the work publicly available in PubMed Central.

Canadian Institutes of Health Research (CIHR to MT; 159727)

Allen Foundation (SLA)

USARMY Department of Defense (SLA and MT; PR171055)

The Polycystic Kidney Disease Foundation USA (MT; 197G14R).

## Supplementary Material

Supplemental data

Unedited blot and gel images

Supporting data values

## Version 1 10/22/2025

Electronic publication

## Version 2 10/29/2025

New supplemental data file: Typographical errors corrected

## References

[1] Danielsen H (1986). Exaggerated natriuresis in adult polycystic kidney disease. Acta Med Scand.

[2] Bankir L, Bichet DG (2012). Polycystic kidney disease: an early urea-selective urine-concentrating defect in ADPKD. Nat Rev Nephrol.

[3] Sullivan LP (1998). Epithelial transport in polycystic kidney disease. Physiol Rev.

[4] Xu N (2006). Autosomal dominant polycystic kidney disease coexisting with cystic fibrosis. J Nephrol.

[5] Terryn S (2011). Fluid transport and cystogenesis in autosomal dominant polycystic kidney disease. Biochim Biophys Acta.

[6] Yang B (2008). Small-molecule CFTR inhibitors slow cyst growth in polycystic kidney disease. J Am Soc Nephrol.

[7] Yanda MK (2018). A potential strategy for reducing cysts in autosomal dominant polycystic kidney disease with a CFTR corrector. J Biol Chem.

[8] Cabrita I (2020). Cyst growth in ADPKD is prevented by pharmacological and genetic inhibition of TMEM16A in vivo. Nat Commun.

[9] Alper SL (2008). Let’s look at cysts from both sides now. Kidney Int.

[10] Lebeau C (2002). Basolateral chloride transporters in autosomal dominant polycystic kidney disease. Pflugers Arch.

[11] Devor DC, Frizzell RA (1993). Calcium-mediated agonists activate an inwardly rectified K+ channel in colonic secretory cells. Am J Physiol.

[12] Devor DC (1996). Modulation of Cl- secretion by benzimidazolones. I. Direct activation of a Ca(2+)-dependent K+ channel. Am J Physiol.

[13] Schwab A (1994). Oscillating activity of a Ca(2+)-sensitive K+ channel. A prerequisite for migration of transformed Madin-Darby canine kidney focus cells. J Clin Invest.

[14] Vandorpe DH (1998). cDNA cloning and functional characterization of the mouse Ca2+-gated K+ channel, mIK1. Roles in regulatory volume decrease and erythroid differentiation. J Biol Chem.

[15] Ishii TM (1997). A human intermediate conductance calcium-activated potassium channel. Proc Natl Acad Sci U S A.

[16] Gerlach AC (2001). ATP-dependent activation of the intermediate conductance, Ca2+-activated K+ channel, hIK1, is conferred by a C-terminal domain. J Biol Chem.

[17] Gerlach AC (2000). Kinase-dependent regulation of the intermediate conductance, calcium-dependent potassium channel, hIK1. J Biol Chem.

[18] Gnanasambandam R (2018). Increased red cell KCNN4 activity in sporadic hereditary xerocytosis associated with enhanced single channel pressure sensitivity of PIEZO1 mutant V598M. Hemasphere.

[19] Shmukler BE (2019). Combined genetic disruption of K-Cl cotransporters and Gardos channel KCNN4 rescues erythrocyte dehydration in the SAD mouse model of sickle cell disease. Blood Cells Mol Dis.

[20] Andolfo I (2013). Multiple clinical forms of dehydrated hereditary stomatocytosis arise from mutations in PIEZO1. Blood.

[21] Zarychanski R (2012). Mutations in the mechanotransduction protein PIEZO1 are associated with hereditary xerocytosis. Blood.

[22] Brugnara C (1993). Inhibition of Ca(2+)-dependent K+ transport and cell dehydration in sickle erythrocytes by clotrimazole and other imidazole derivatives. J Clin Invest.

[23] De Franceschi L (1994). Treatment with oral clotrimazole blocks Ca(2+)-activated K+ transport and reverses erythrocyte dehydration in transgenic SAD mice. A model for therapy of sickle cell disease. J Clin Invest.

[24] Ataga KI (2008). Efficacy and safety of the Gardos channel blocker, senicapoc (ICA-17043), in patients with sickle cell anemia. Blood.

[25] Begenisich T (2004). Physiological roles of the intermediate conductance, Ca2+-activated potassium channel Kcnn4. J Biol Chem.

[26] Si H (2006). Impaired endothelium-derived hyperpolarizing factor-mediated dilations and increased blood pressure in mice deficient of the intermediate-conductance Ca2+-activated K+ channel. Circ Res.

[27] Di L (2010). Inhibition of the K+ channel KCa3.1 ameliorates T cell-mediated colitis. Proc Natl Acad Sci U S A.

[28] Pang CJ (2012). Kruppel-like factor 1 (KLF1), KLF2, and Myc control a regulatory network essential for embryonic erythropoiesis. Mol Cell Biol.

[29] Lanoix J (1996). Dysregulation of cellular proliferation and apoptosis mediates human autosomal dominant polycystic kidney disease (ADPKD). Oncogene.

[30] Trudel M (1991). C-myc as an inducer of polycystic kidney disease in transgenic mice. Kidney Int.

[31] Parrot C (2019). c-Myc is a regulator of the PKD1 gene and PC1-induced pathogenesis. Hum Mol Genet.

[32] Albaqumi M (2008). KCa3.1 potassium channels are critical for cAMP-dependent chloride secretion and cyst growth in autosomal-dominant polycystic kidney disease. Kidney Int.

[33] Shibazaki S (2008). Cyst formation and activation of the extracellular regulated kinase pathway after kidney specific inactivation of Pkd1. Hum Mol Genet.

[34] Yu S (2007). Essential role of cleavage of Polycystin-1 at G protein-coupled receptor proteolytic site for kidney tubular structure. Proc Natl Acad Sci U S A.

[35] Kurbegovic A (2014). Novel functional complexity of polycystin-1 by GPS cleavage in vivo: role in polycystic kidney disease. Mol Cell Biol.

[36] Kurbegovic A (2010). Pkd1 transgenic mice: adult model of polycystic kidney disease with extrarenal and renal phenotypes. Hum Mol Genet.

[37] Thivierge C (2006). Overexpression of PKD1 causes polycystic kidney disease. Mol Cell Biol.

[38] Ward CJ (1996). Polycystin, the polycystic kidney disease 1 protein, is expressed by epithelial cells in fetal, adult, and polycystic kidney. Proc Natl Acad Sci U S A.

[39] Weston BS (1997). Polycystin expression during embryonic development of human kidney in adult tissues and ADPKD tissue. Histochem J.

[40] Palsson R (1996). Characterization and cell distribution of polycystin, the product of autosomal dominant polycystic kidney disease gene 1. Mol Med.

[41] Geng L (1996). Identification and localization of polycystin, the PKD1 gene product. J Clin Invest.

[42] Torres VE (2019). Pro: tolvaptan delays the progression of autosomal dominant polycystic kidney disease. Nephrol Dial Transplant.

[43] Hogan MC (2020). Pansomatostatin agonist pasireotide long-acting release for patients with autosomal dominant polycystic kidney or liver disease with severe liver involvement: a randomized clinical trial. Clin J Am Soc Nephrol.

[44] Kraus A (2018). HIF-1α promotes cyst progression in a mouse model of autosomal dominant polycystic kidney disease. Kidney Int.

[45] Davidow CJ (1996). The cystic fibrosis transmembrane conductance regulator mediates transepithelial fluid secretion by human autosomal dominant polycystic kidney disease epithelium in vitro. Kidney Int.

[46] Venkatesan V (2024). The role of Aquaporin-2 in cyst progression in autosomal dominant polycystic kidney disease. Proc Physiol Soc.

[47] Magenheimer BS (2006). Early embryonic renal tubules of wild-type and polycystic kidney disease kidneys respond to cAMP stimulation with cystic fibrosis transmembrane conductance regulator/Na(+),K(+),2Cl(-) Co-transporter-dependent cystic dilation. J Am Soc Nephrol.

[48] Ataga KI (2011). Improvements in haemolysis and indicators of erythrocyte survival do not correlate with acute vaso-occlusive crises in patients with sickle cell disease: a phase III randomized, placebo-controlled, double-blind study of the Gardos channel blocker senicapoc (ICA-17043). Br J Haematol.

[49] Jin LW (2019). Repurposing the KCa3.1 inhibitor senicapoc for Alzheimer’s disease. Ann Clin Transl Neurol.

[50] Wulff H (2000). Design of a potent and selective inhibitor of the intermediate-conductance Ca2+-activated K+ channel, IKCa1: a potential immunosuppressant. Proc Natl Acad Sci U S A.

[51] Buchholz B (2014). Anoctamin 1 induces calcium-activated chloride secretion and proliferation of renal cyst-forming epithelial cells. Kidney Int.

[52] Tradtrantip L (2009). Nanomolar potency pyrimido-pyrrolo-quinoxalinedione CFTR inhibitor reduces cyst size in a polycystic kidney disease model. J Med Chem.

[53] Harris PC, Torres VE (2014). Genetic mechanisms and signaling pathways in autosomal dominant polycystic kidney disease. J Clin Invest.

[54] Yamaguchi T (2004). Calcium restriction allows cAMP activation of the B-Raf/ERK pathway, switching cells to a cAMP-dependent growth-stimulated phenotype. J Biol Chem.

[55] Besschetnova TY (2010). Identification of signaling pathways regulating primary cilium length and flow-mediated adaptation. Curr Biol.

[56] de Almeida RM (2016). Transcriptome analysis reveals manifold mechanisms of cyst development in ADPKD. Hum Genomics.

[57] Muto Y (2021). Single cell transcriptional and chromatin accessibility profiling redefine cellular heterogeneity in the adult human kidney. Nat Commun.

[58] Chen L (2021). A comprehensive map of mRNAs and their isoforms across all 14 renal tubule segments of mouse. J Am Soc Nephrol.

[59] Chen L (2017). Transcriptomes of major renal collecting duct cell types in mouse identified by single-cell RNA-seq. Proc Natl Acad Sci U S A.

[60] Clark JZ (2019). Representation and relative abundance of cell-type selective markers in whole-kidney RNA-seq data. Kidney Int.

[61] Janssen P (2023). The effect of background noise and its removal on the analysis of single-cell expression data. Genome Biol.

[62] Wu H (2019). Advantages of single-nucleus over single-cell RNA sequencing of adult kidney: rare cell types and novel cell states revealed in fibrosis. J Am Soc Nephrol.

[63] Lee JW (2015). Deep sequencing in microdissected renal tubules identifies nephron segment–specific transcriptomes. J Am Soc Nephrol.

[64] Silic MR (2021). Phylogenetic and developmental analyses indicate complex functions of calcium-activated potassium channels in zebrafish embryonic development. Dev Dyn.

[65] Rutledge EA (2017). Cellular heterogeneity in the ureteric progenitor niche and distinct profiles of branching morphogenesis in organ development. Development.

[66] Howden SE (2021). Plasticity of distal nephron epithelia from human kidney organoids enables the induction of ureteric tip and stalk. Cell Stem Cell.

[67] Trudel M (1998). Polycystic kidney disease in SBM transgenic mice: role of c-myc in disease induction and progression. Am J Pathol.

[68] Barisoni L (1995). Analysis of the role of membrane polarity in polycystic kidney disease of transgenic SBM mice. Am J Pathol.

[69] Trudel M, D’Agati V (1992). A model of polycystic kidney disease in SBM transgenic mice. Contrib Nephrol.

[70] Elberg G (2008). Plasticity of epithelial cells derived from human normal and ADPKD kidneys in primary cultures. Cell Tissue Res.

[71] Thomson RB (2003). Histopathological analysis of renal cystic epithelia in the Pkd2WS25/- mouse model of ADPKD. Am J Physiol Renal Physiol.

[72] Belibi FA (2004). Cyclic AMP promotes growth and secretion in human polycystic kidney epithelial cells. Kidney Int.

[73] Yamaguchi T (2006). Calcium restores a normal proliferation phenotype in human polycystic kidney disease epithelial cells. J Am Soc Nephrol.

[74] Wong R, Schlichter LC (2014). PKA reduces the rat and human KCa3.1 current, CaM binding, and Ca2+ signaling, which requires Ser332/334 in the CaM-binding C terminus. J Neurosci.

[75] Schmid D (2014). Transcriptional regulation induced by cAMP elevation in mouse Schwann cells. ASN Neuro.

[76] Yamaguchi T (2000). cAMP stimulates the in vitro proliferation of renal cyst epithelial cells by activating the extracellular signal-regulated kinase pathway. Kidney Int.

[77] Stork PJ, Schmitt JM (2002). Crosstalk between cAMP and MAP kinase signaling in the regulation of cell proliferation. Trends Cell Biol.

[78] Tsai WB (2012). Activation of Ras/PI3K/ERK pathway induces c-Myc stabilization to upregulate argininosuccinate synthetase, leading to arginine deiminase resistance in melanoma cells. Cancer Res.

[79] Zuo Z (2023). ERK and c-Myc signaling in host-derived tumor endothelial cells is essential for solid tumor growth. Proc Natl Acad Sci U S A.

[80] Kurbegovic A, Trudel M (2020). The master regulators Myc and p53 cellular signaling and functions in polycystic kidney disease. Cell Signal.

[81] Mills JC (2019). Nomenclature for cellular plasticity: are the terms as plastic as the cells themselves?. EMBO J.

[82] Assmus AM (2020). Cellular plasticity: a mechanism for homeostasis in the kidney. Acta Physiol (Oxf).

[83] Grgic I (2009). Renal fibrosis is attenuated by targeted disruption of KCa3.1 potassium channels. Proc Natl Acad Sci U S A.

[84] Huang C (2013). Blockade of KCa3.1 ameliorates renal fibrosis through the TGF-β1/Smad pathway in diabetic mice. Diabetes.

[85] Chen CL (2016). Blockade of KCa3.1 potassium channels protects against cisplatin-induced acute kidney injury. Arch Toxicol.

[86] Paka L (2017). Anti-steatotic and anti-fibrotic effects of the KCa3.1 channel inhibitor, Senicapoc, in non-alcoholic liver disease. World J Gastroenterol.

[87] Organ L (2017). Inhibition of the K_Ca_3.1 channel alleviates established pulmonary fibrosis in a large animal model. Am J Respir Cell Mol Biol.

[88] Xie H (2018). The KCa3.1 blocker TRAM-34 inhibits proliferation of fibroblasts in paraquat-induced pulmonary fibrosis. Toxicol Lett.

[89] Srivastava S (2006). Phosphatidylinositol-3 phosphatase myotubularin-related protein 6 negatively regulates CD4 T cells. Mol Cell Biol.

[90] Kohler R (2003). Blockade of the intermediate-conductance calcium-activated potassium channel as a new therapeutic strategy for restenosis. Circulation.

[91] Wulff H (2004). K+ channel expression during B cell differentiation: implications for immunomodulation and autoimmunity. J Immunol.

[92] Pinto CS (2012). Calmodulin-sensitive adenylyl cyclases mediate AVP-dependent cAMP production and Cl- secretion by human autosomal dominant polycystic kidney cells. Am J Physiol Renal Physiol.

[93] Hopp K (2012). Functional polycystin-1 dosage governs autosomal dominant polycystic kidney disease severity. J Clin Invest.

[94] Brown BM (2020). Pharmacology of small- and intermediate-conductance calcium-activated potassium channels. Annu Rev Pharmacol Toxicol.

[95] Kanhai AA (2020). Renal cyst growth is attenuated by a combination treatment of tolvaptan and pioglitazone, while pioglitazone treatment alone is not effective. Sci Rep.

[96] Cao X (2022). Benzodiazepine derivatives as potent vasopressin V_2_ receptor antagonists for the treatment of autosomal dominant kidney disease. J Med Chem.

[97] Gattone VH (2003). Inhibition of renal cystic disease development and progression by a vasopressin V2 receptor antagonist. Nat Med.

[98] Hopp K (2015). Tolvaptan plus pasireotide shows enhanced efficacy in a PKD1 model. J Am Soc Nephrol.

[99] Bachinsky DR (1995). Water channel expression in human ADPKD kidneys. Am J Physiol.

[100] Ataga KI (2021). Haemoglobin response to senicapoc in patients with sickle cell disease: a re-analysis of the phase III trial. Br J Haematol.

[101] Wu G (2002). Trans-heterozygous Pkd1 and Pkd2 mutations modify expression of polycystic kidney disease. Hum Mol Genet.

[102] Piontek KB (2004). A functional floxed allele of Pkd1 that can be conditionally inactivated in vivo. J Am Soc Nephrol.

[103] Shao X (2002). Epithelial-specific Cre/lox recombination in the developing kidney and genitourinary tract. J Am Soc Nephrol.

[104] Kurbegovic A (2023). Modeling Pkd1 gene-targeted strategies for correction of polycystic kidney disease. Mol Ther Methods Clin Dev.

[105] Bankhead P (2017). QuPath: open source software for digital pathology image analysis. Sci Rep.

