# Supplementary material for: Kcnn4/KCa3.1 inhibition blunts polycystic kidney disease progression in mouse models
Source: JCI Insight. 2025 Oct 22;10(20):e191311. doi: 10.1172/jci.insight.191311 (PMC12581680; doi:10.1172/jci.insight.191311)
Supplement: Supplemental data [file jciinsight-10-191311-s293.pdf]

***Kcnn4*/Kca3.1 inhibition blunts polycystic kidney disease progression in mouse models**

Short title : **Senicapoc treatment slows progression of PKD**

Supplemental data

# Figure S1

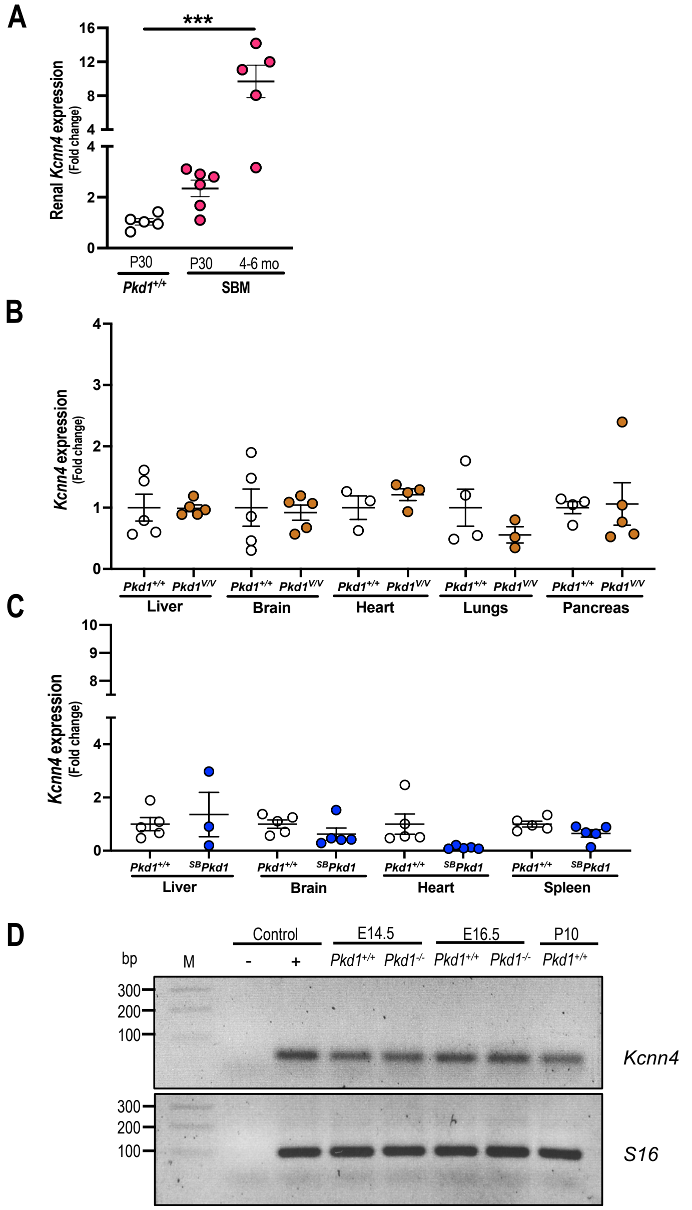


**Figure S1. Characterization of *Kcnn4* expression in renal and extrarenal tissues.**

A. Relative expression (qPCR) of *Kcnn4* in 1 mo and 4-6mo kidneys of transgenic SBM mice (pink circles) vs 1 mo $Pkd1^{+/+}$ kidneys (open circles). ***, p<0.001, ANOVA.

B. Relative expression (qPCR) of *Kcnn4* in P10 extrarenal tissues of $Pkd1^{V/V}$ mice (caramel circles) vs $Pkd1^{+/+}$ mice (open circles).

C. Relative expression (qPCR) of *Kcnn4* in P10 extrarenal tissues of $^{SB}Pkd1$ mice (blue circles).

D. Expression of *Kcnn4* (58bp) detected by PCR in E14.5 and E16.5 $Pkd1^{+/+}$ and $Pkd1^{-/-}$ embryonic kidneys/ metanephroi and in P10 WT kidneys. Ribosomal S16 (103bp) served as internal control.

## Figure S2

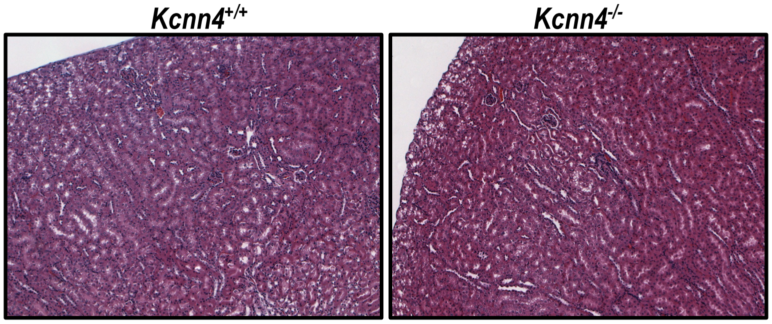


**Figure S2. Normal renal histology of *Kcnn4*$^{-/-}$ mice.**

Representative kidney sections of adult *Kcnn4*$^{-/-}$ and age-matched control *Kcnn4*$^{+/+}$ mice (H&E).

# Figure S3

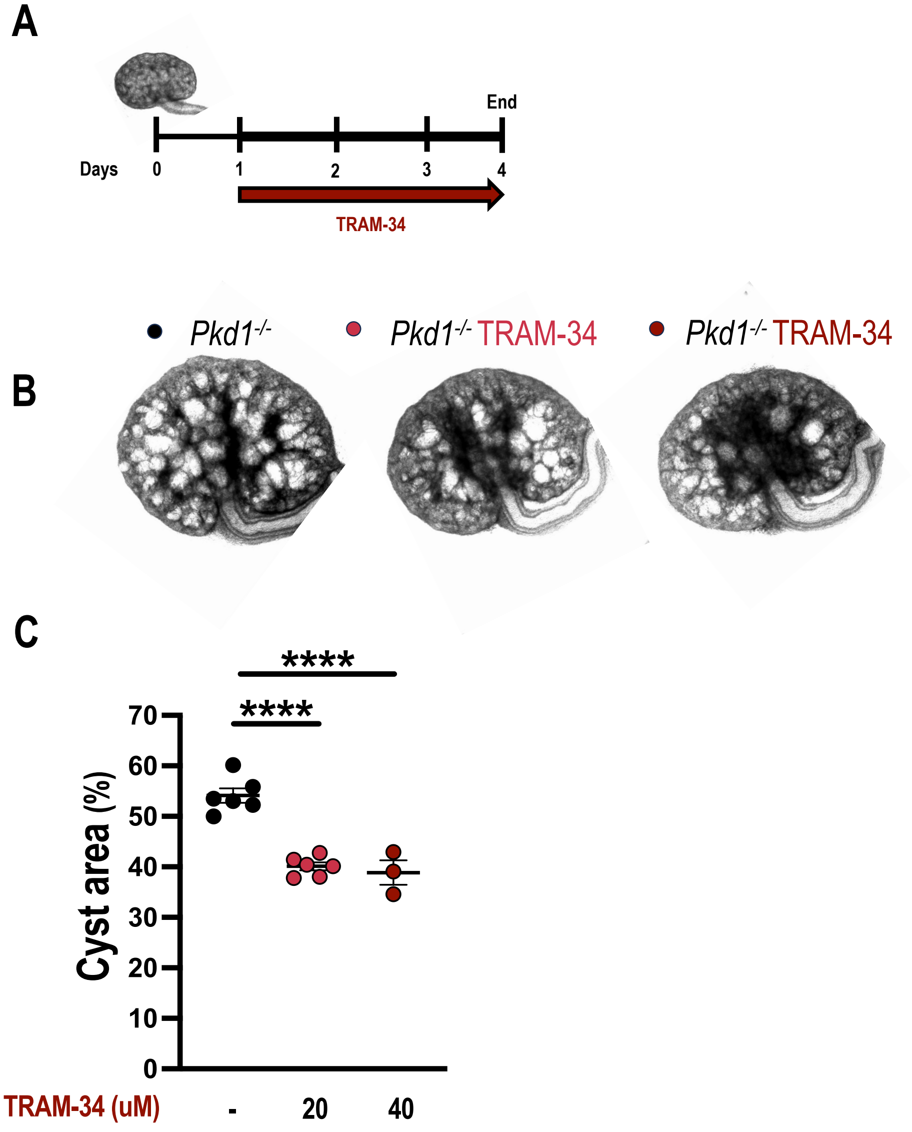


**Figure S3. KCa3.1 inhibitor TRAM-34 delays cyst growth in *Pkd1*$^{-/-}$ metanephroi.**

A. Experimental design: metanephroi were stimulated with cAMP (thick line) from day 1 in the absence or presence of 20µM TRAM-34 (dark red arrow).

B. Representative day 4 images of *Pkd1*$^{-/-}$ metanephroi in the absence or presence of TRAM-34 at 20µM (brick red circles) and 40µM (dark brick circles).

C. Cyst area (%) of day 4 *Pkd1*$^{-/-}$ metanephroi treated with vehicle (black circles) or with 20µM (brick circles) or 40µM TRAM-34 (dark brick circles). ****, $p<0.0001$, ANOVA.

## Figure S4

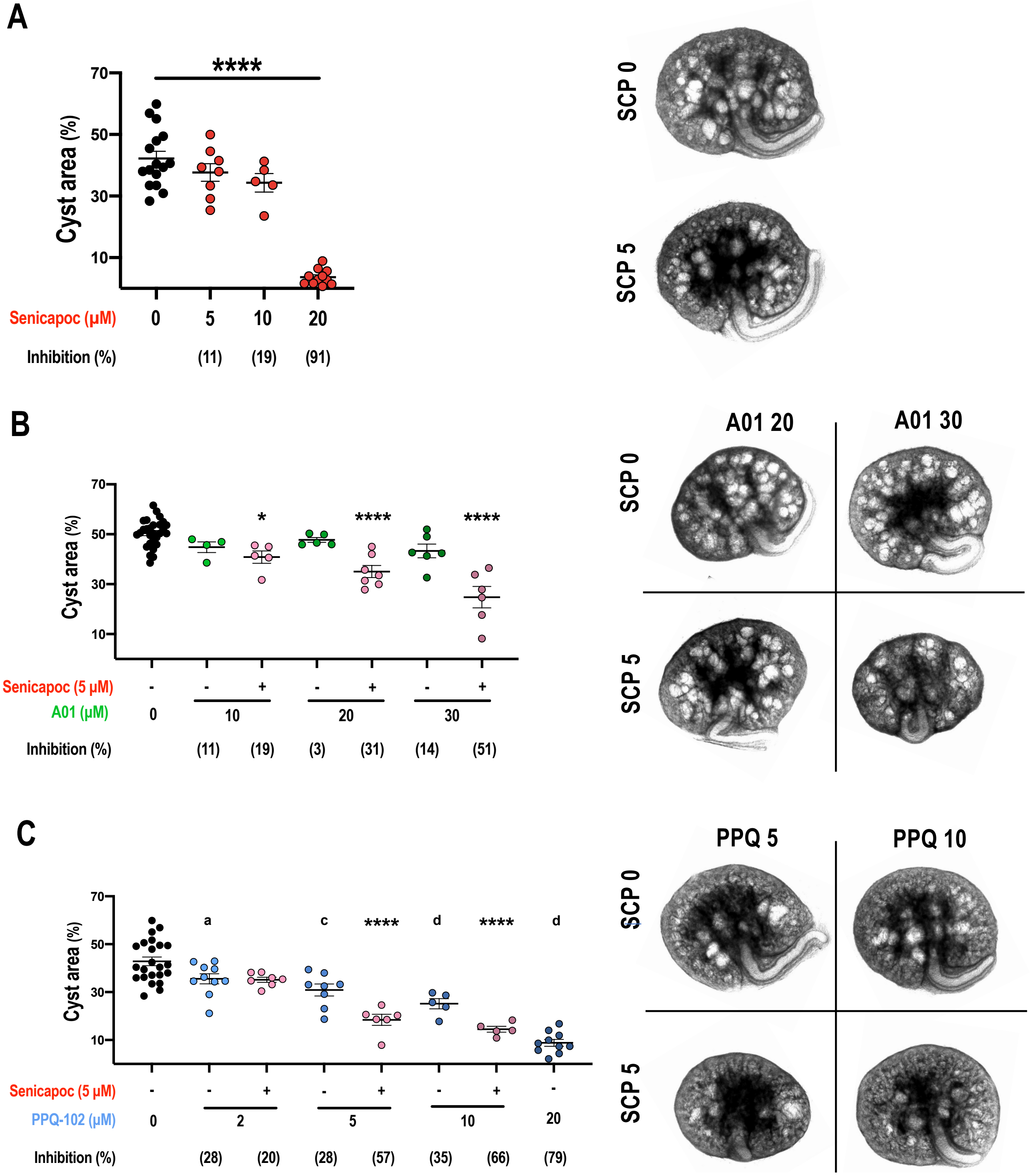


**Figure S4. Senicapoc inhibition of cyst growth is potentiated by inhibitors of TMEM16A and of CFTR.**

A. Cyst area (%) of *Pkd1*$^{-/-}$ metanephroi treated with vehicle (black circles) or with senicapoc (SCP) at 5, 10 or 20µM (red circles). Right, representative images of day 4 metanephroi. ****, p<0.0001, ANOVA.

B. Cyst area (%) of *Pkd1*$^{-/-}$ metanephroi treated with vehicle (black circles) or with TMEM16A inhibitor CaCCin-A01 (A01) (green circles of intensity proportional to A01 concentration) in the absence or presence of 5µM SCP (pink circles of intensity proportional to A01 concentration). Right, representative images of day 4 metanephroi treated with A01 in presence or absence of SCP. *, p<0.05, ****, p<0.0001, ANOVA.

C. Cyst area (%) of *Pkd1*$^{-/-}$ metanephroi treated with vehicle (black circles) or with CFTR inhibitor PPQ-102 (blue circles of intensity proportional to PPQ-102 concentration) in the absence or presence of 5µM SCP (pink circles of intensity proportional to PPQ-102 concentration). Right, representative images of day 4 metanephroi treated with PPQ-102 in presence or absence of SCP. Statistical comparisons of *Pkd1*$^{-/-}$ with PPQ-102 alone [a], p<0.05; [c], p<0.001; [d], p<0.0001 or with PPQ-102 plus SCP ****, p<0.0001, ANOVA.

## Figure S5

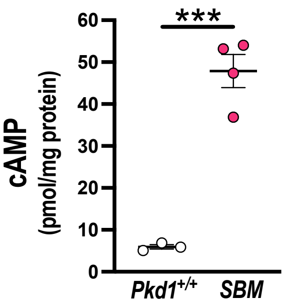


**Figure S5. Increased cAMP levels in SBM mouse model.**

Renal cAMP levels in 2.5-4.5 months wild-type *Pkd1*$^{+/+}$ (open circles) and *SBM* (pink circles) mice. ***, p<0.001, Student's t-test, one tail.

# Figure S6

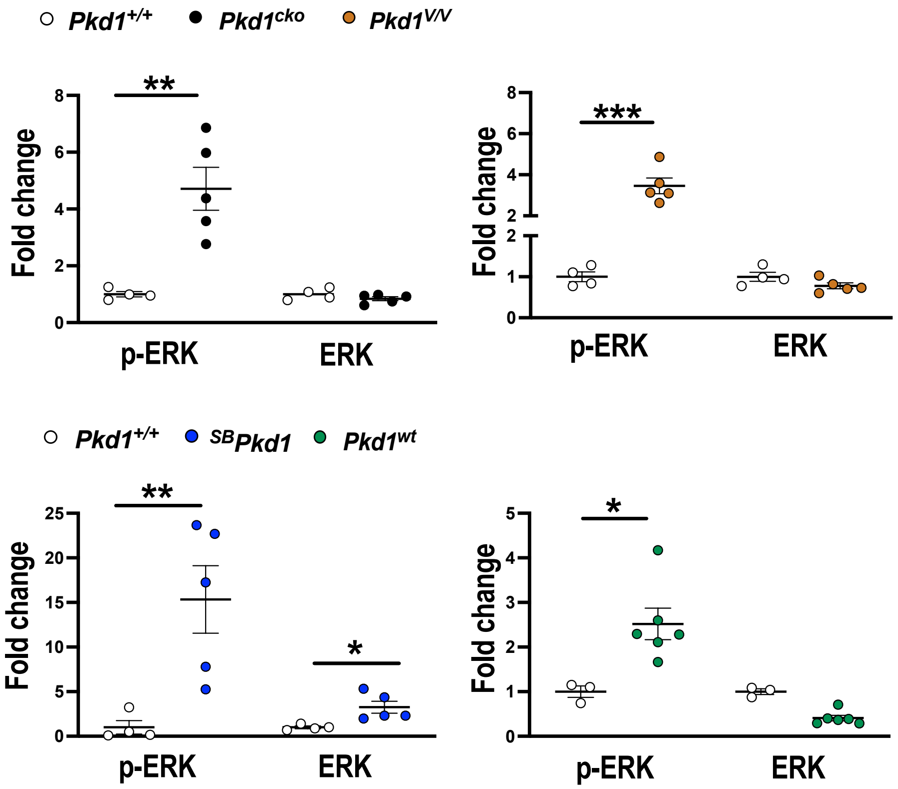


**Figure S6. Increased p-ERK in *Pkd1* mouse models.**

Densitometric measurements of GAPDH-normalized levels of pERK and ERK in (upper panels) kidneys from *Pkd1*$^{+/+}$ mice (open circles), more rapidly progressive ADPKD mouse models *Pkd1*$^{cko}$ (black circles) and *Pkd1*$^{V/V}$ (caramel circles), and (bottom panels) from slowly progressive models $^{SB}$*Pkd1*(blue circles) and *Pkd1*$^{wt}$ (green circles) *, p<0.05; **, p<0.01; ***, p<0.001, Student's t-test, one tail.

# Figure S7

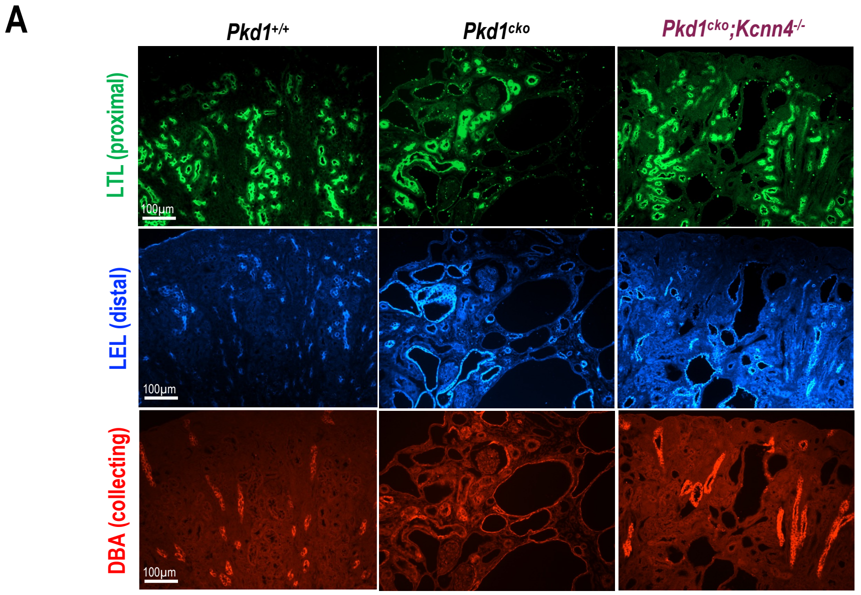


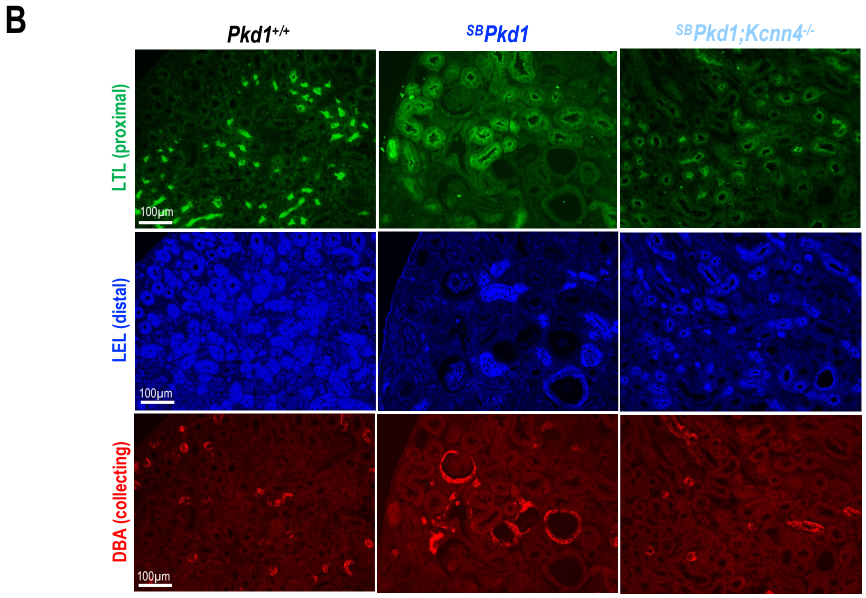


**Figure S7. Attenuation of individual cystic nephron segments in ADPKD mouse models by genetic inactivation of *Kcnn4*.**

A. Nephron segment origins of cystic tubules in kidney sections from representative P5 *Pkd1*$^{+/+}$, *Pkd1*$^{cko}$ and *Pkd1*$^{cko}$*;Kcnn4*$^{-/-}$ mice assessed by immunofluorescence staining with segment-specific lectin markers LTL (upper row, proximal tubule, green), LEL (middle row, distal tubule, blue) and DBA (lower row, collecting duct, red).

B. Nephron segment origins of cystic tubules in kidney sections from representative 2 mo *Pkd1*$^{+/+}$, $^{SB}$*Pkd1* and $^{SB}$*Pkd1;Kcnn4*$^{-/-}$ mice assessed by fluorescence staining with segment-specific lectin markers LTL (upper row, proximal tubule, green), LEL (middle row, distal tubule, blue) and DBA (lower row, collecting duct, red).

# Figure S8

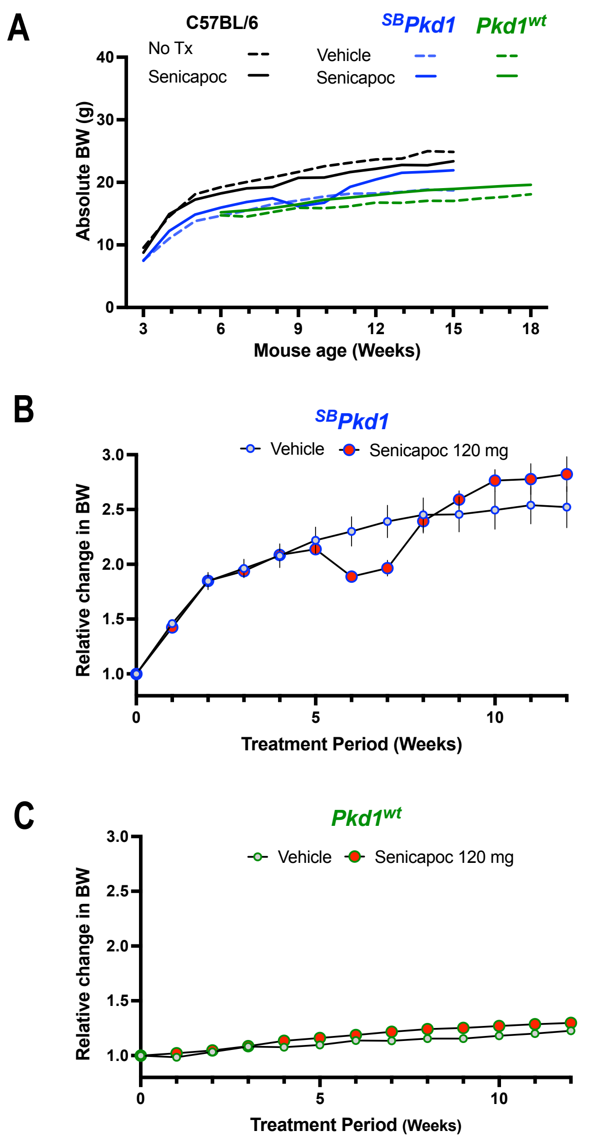


**Figure S8. Absolute and relative body weight changes in senicapoc-treated and -untreated mice.**

A. Absolute body weight (BW) of control C57BL/6J mice (black) $^{SB}$*Pkd1* (blue) and *Pkd1*$^{wt}$ mice (green) was monitored during administration of senicapoc (solid line) or vehicle (stippled lines), or without treatment (no Tx, stippled line).

B. Relative BW change of $^{SB}$*Pkd1* mice during administration of vehicle (blue-rimmed for genotype, grey-filled circles) or 120 mg/kg senicapoc (blue-rimmed, red-filled circles) from age 3 wks . Relative BW change +SEM is BW at start of experiment normalized to 1.0, and subsequent normalized weights represent later-dated BWs divided by starting BW.

C. Relative BW change +SEM of *Pkd1*$^{wt}$ mice during administration of vehicle (green-rimmed for genotype, grey-filled circles) or 120 mg/kg senicapoc (green-rimmed, red-filled circles) from age 6 wks.

**Supplemental Table 1** Primers for genotyping

| | | |
|---|---|---|
| *KspCre* | Forward | 5’ AGG TTC GTG CAC TCA TGG A 3’ |
| | Reverse | 5’ TCG ACC AGT TTA GTT ACC C 3’ |
| *Pkd1$^{flox}$* | Forward | 5’ CCT GCC TTG CTC TAC TTT CC 3’ |
| | Reverse | 5’ AGG GCT TTT CTT GCT GGT CT 3’ |
| *Kcnn4* | Forward | 5’ TTG GTG TGC TCA GAC CTG CTG 3’ |
| | Reverse | 5’ GAG CTC ACG CAG TCA CAC AT 3’ |
| *Pkd1$^{+/-}$* | Forward | 5’ CCC TCC TGA ACT GCG GCT 3’ |
| | Forward | 5’ AGC GCA TCG CCT TCT ATC GC 3’ |
| | Reverse | 5’ CAG GGT CTC CGG CCA G 3’ |
| *Pkd1$^{v}$* | Forward | 5’ CCA AAC AAC TCA GAC CAG G 3’ |
| | Reverse | 5’ ACC AGG ACA GCA AGA AAA C 3’ |
| *Pkd1 (*exon 7) | Forward | 5’ TGG TAC CTG ATT GGG CAT GAT 3’ |
| *Pkd1* (exon 15) | Reverse | 5’ GTT TTG CCT GGA TCC GCT GTT G 3’ |

**Supplemental Table 2** Primers for qPCR

| | | |
|---|---|---|
| *Kcnn4* | Forward | 5’ CGT GCA CAA CTT CAT GAT GGA 3’ |
| | Reverse | 5’ CGC CGC TGA CTC CTT CA 3’ |
| *Aqp1* | Forward | 5’ CAT TTG GCT CTG CTG TGC TC 3’ |
| | Reverse | 5’ TGA TGT CGT CAG CAT CCA GG 3’ |
| *Aqp2* | Forward | 5’ CCA TTG GTT TCT CTG TTA CCC TG 3’ |
| | Reverse | 5’ CGG TGA AAT AGA TCC CAA GGA G 3’ |
| *Nkcc1* | Forward | 5’ GGA TGG CTT TGC GAA TGG AG 3’ |
| | Reverse | 5’ TGC AGC GGA CTA ATA CAC CC 3’ |
| *Nkcc2* | Forward | 5’ CTG GCC TCA TAT GCG CTT ATT 3’ |
| | Reverse | 5’ AGA TTT GGC ATA CGA GGC ATG 3’ |
| *Cftr* | Forward | 5’ CGG CGA TGC TTT TTC TGG AG 3’ |
| | Reverse | 5’ TGG GTG AAG AAG CAG TGT CC 3’ |
| *Ano1* | Forward | 5’ TGA GGG TGA CAA CGT TGA GTT C 3’ |
| | Reverse | 5’ CGT AAC TTG CCC ATT CCT CAT AC 3’ |
| *S16* | Forward | 5’ GCT ACC AGG GCC TTT GAG ATG 3’ |
| | Reverse | 5’ AGG AGC GAT TTG CTG GTG TGG 3’ |
| *Actin* | Forward | 5’ TAT TGG CAA CGA GCG GTT CC 3’ |
| | Reverse | 5’ GGC ATA GAG GTC TTT ACG GAT GTC 3’ |
| *Hprt1* | Forward | 5’ GGC CAG ACT TTG TTG GAT TTG 3’ |
| | Reverse | 5’ TGC GCT CAT CTT AGG CTT TGT 3’ |
| *Gapdh* | Forward | 5’ AAG GTC ATC CCA GAG CTG AA 3’ |
| | Reverse | 5’ CTG CTT CAC CAC CTT CTT GA 3’ |
| *KCNN4* | | Taqman # 4453320 (FAM-MGB) |
| *HPRT1* | Forward | 5’ GAA AAG GAC CCC ACG AAG TGT 3’ |
| | Reverse | 5’ AGT CAA GGG CAT ATC CTA CAA CA 3’ |

**Supplemental Table 3** Detailed antibody information

| Experiment | Primary/Secondary | Antibody/Ligand | Dilution | Company | ID |
|---|---|---|---|---|---|
| ***Immunoblotting*** | | | | | |
| | Primary | Rabbit Anti-c-Myc | 1:1000 | Abcam | ab32072 |
| | Primary | Mouse anti-phospho-Erk1/2 | 1:1000 | Cell Signaling | 9106 |
| | Primary | Rabbit anti-Erk1/2 | 1:1000 | Cell Signaling | 4695 |
| | Primary | Mouse Anti-GAPDH | 1:10000 | Abcam | ab8245 |
| | Secondary | Rabbit Anti-Mouse IgG, Peroxidase | 1:5000 | Sigma | A9044 |
| | Secondary | Goat Anti-Rabbit IgG, Peroxidase | 1:2000 | Sigma | A0545 |
| ***Immunofluorescence*** | | | | | |
| Tubule cyst PROXIMAL | Primary conjugated | Lotus Tetragonolobus Lectin (LTL), Fluorescein | 1:200 | Vector Laboratories | FL-1321 |
| Tubule cyst COLLECTING | Primary conjugated | Dolichos Biflorus Agglutinin (DBA), Rhodamine | 1:50 | Vector Laboratories | RL-1032 |
| Tubule cyst DISTAL | Primary | Lycopersicon esculentum Lectin (LEL), Biotinylated | 1:100 | Vector Laboratories | B-1175 |
| | Secondary | Streptavidin, AMCA | 1:100 | Vector Laboratories | SA-5008 |
| Cilia measurements | Primary | Mouse Anti-Acetylated $\alpha$-Tubulin | 1:1000 | Sigma | T7451 |
| | Secondary | Goat anti-Mouse IgG (H+L) Alexa Fluor™ 555 | 1:500 | Invitrogen | A28180 |
| Proliferation assay | Primary | Rabbit Anti-ki67 | 1:100 | Leica | NCL-Ki67p |
| | Secondary | Goat Anti-Rabbit IgG (H+L), Biotinylated | 1:300 | Vector Laboratories | BA-1000 |
