# Supplementary material for: Kcnn4/KCa3.1 inhibition blunts polycystic kidney disease progression in mouse models
Source: JCI Insight. 2025 Oct 22;10(20):e191311. doi: 10.1172/jci.insight.191311 (PMC12581680; doi:10.1172/jci.insight.191311)
Supplement: Unedited blot and gel images [file jciinsight-10-191311-s294.pdf]

Kcnn4/Kca3.1 inhibition blunts polycystic kidney disease progression in mouse models

Short title : **Senicapoc treatment slows progression of PKD**

Unedited blots and gel images

Figure 5B

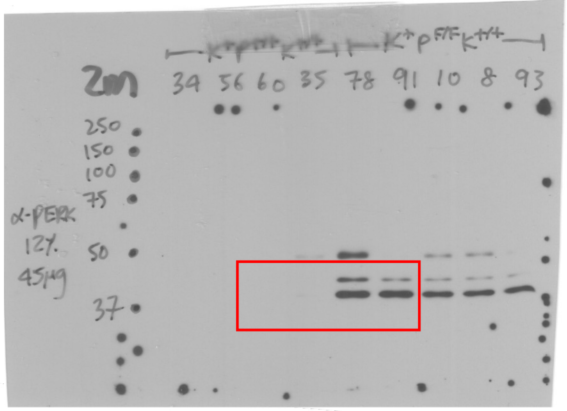

p-ERK

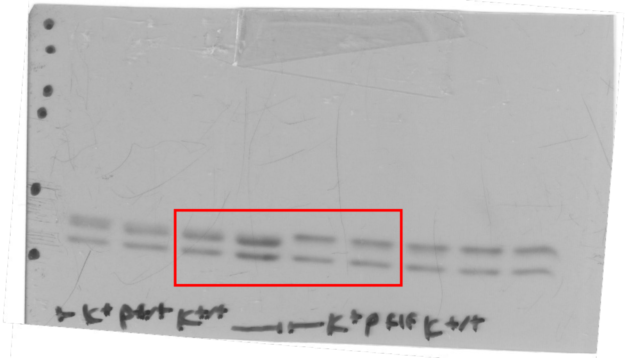

ERK

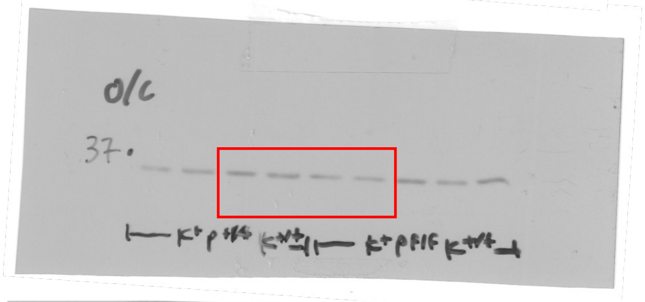

GAPDH

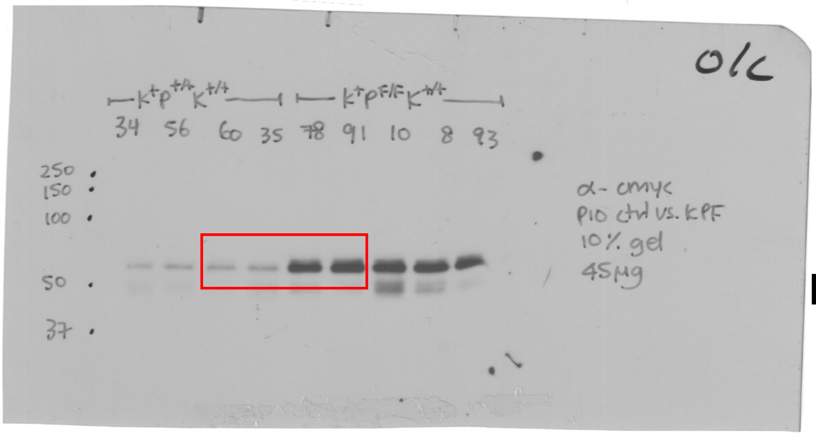

Myc

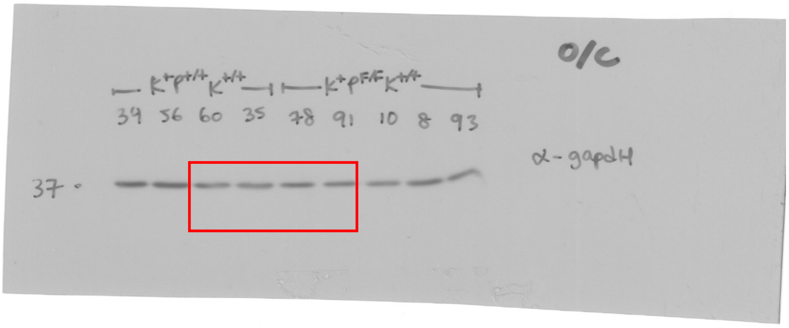

GAPDH

Figure 5C

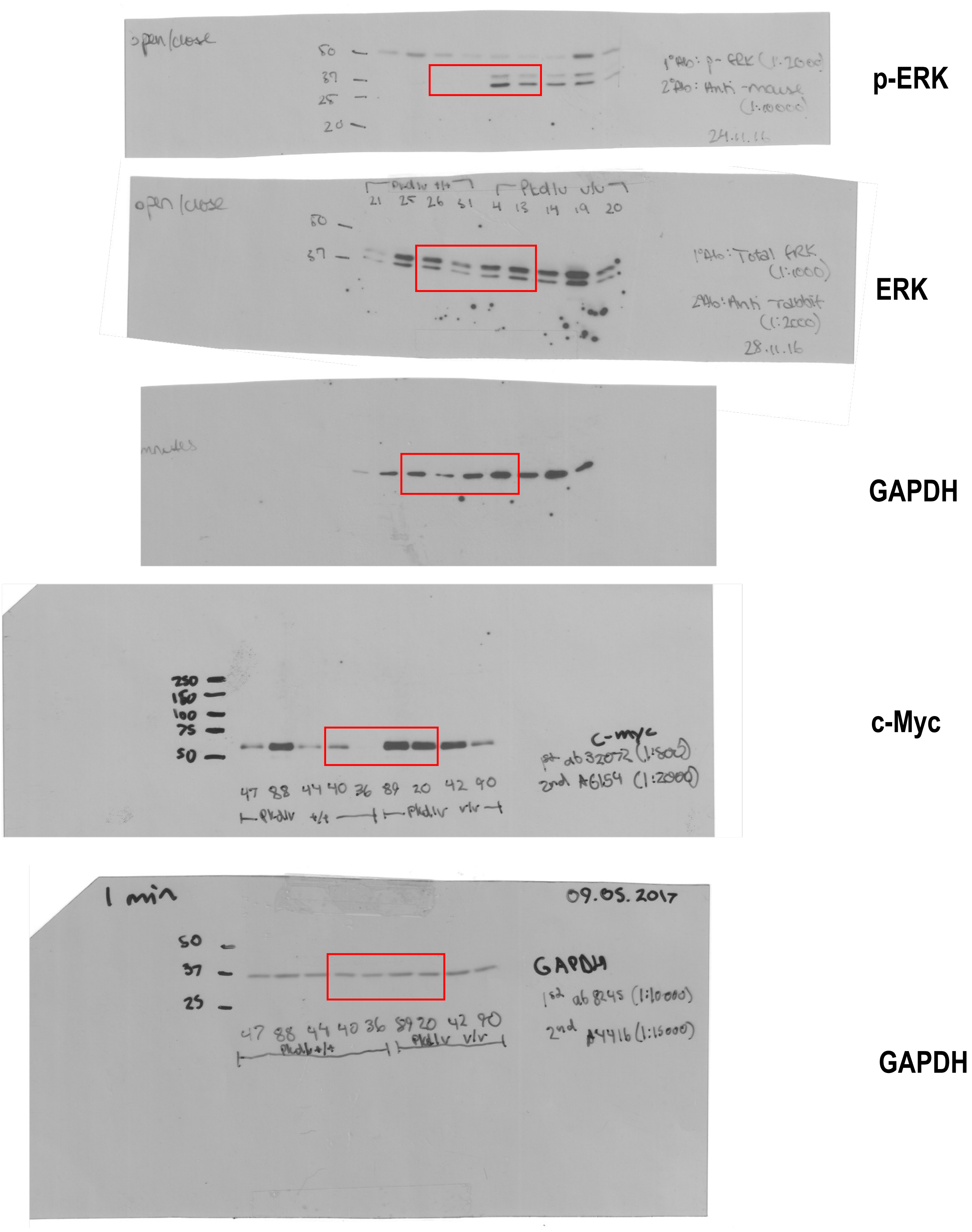
p-ERK
ERK
GAPDH
c-Myc
GAPDH
open/close
24.11.16
28.11.16
c-myc
1 min
09.05.2017
GAPDH

Figure 5D

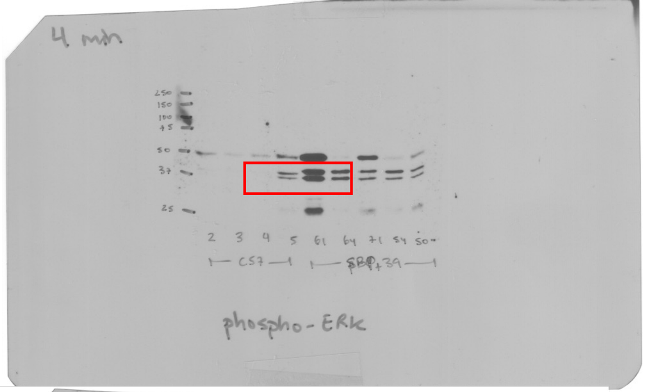

p-ERK

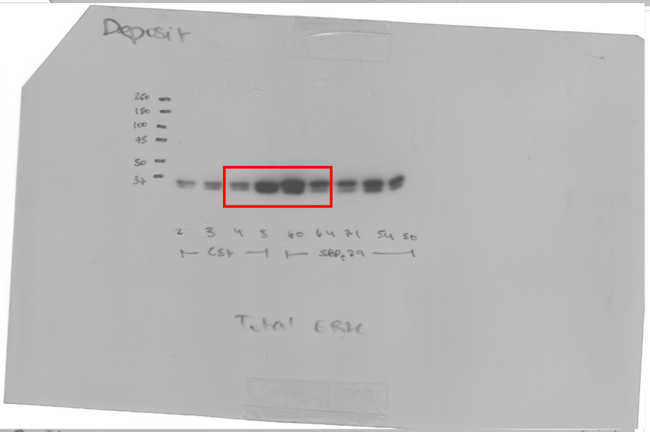

ERK

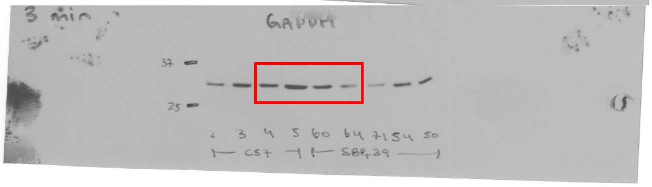

GAPDH

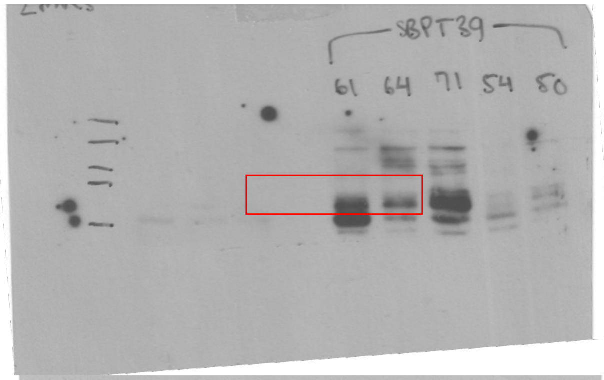

c-Myc

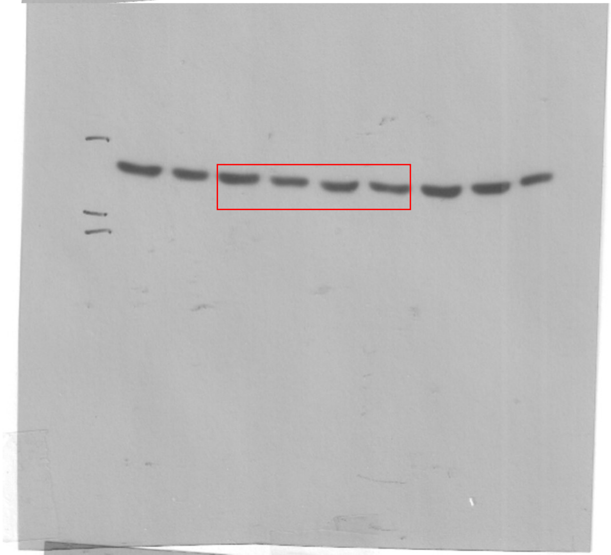

GAPDH

Figure 5E

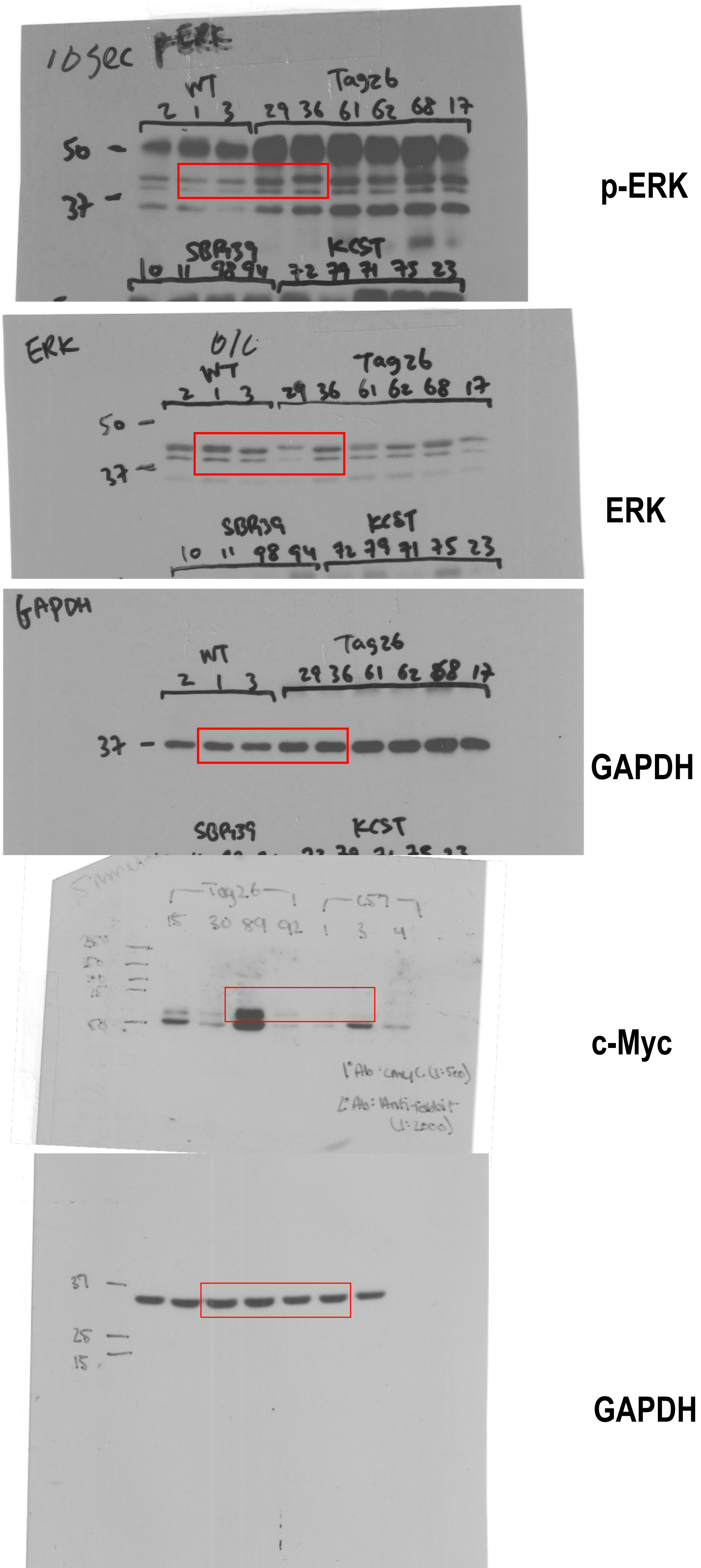

Figure 6J

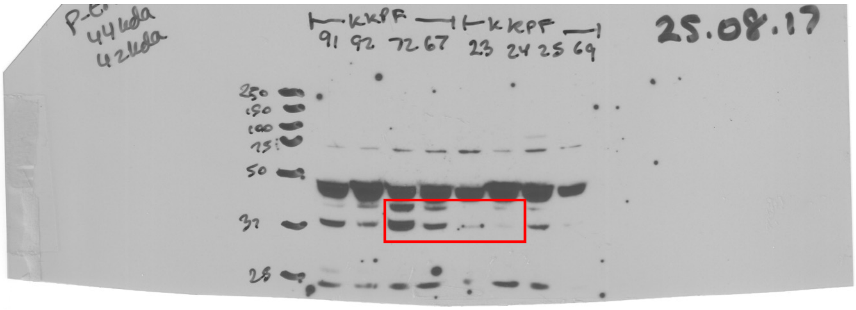

p-ERK

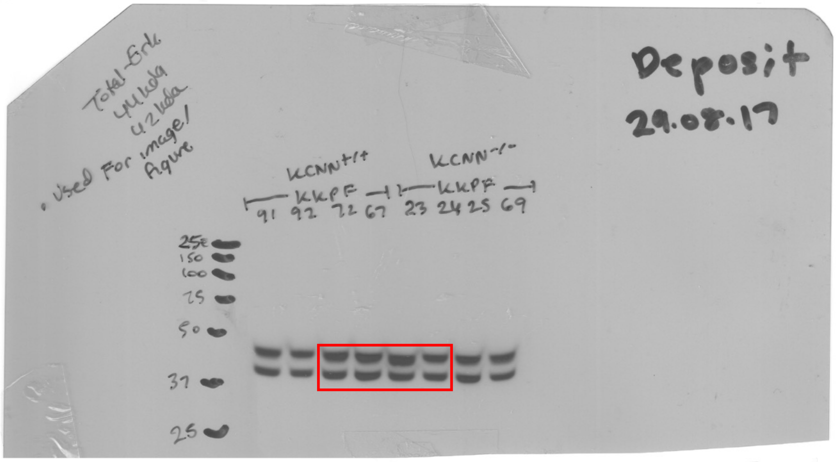

ERK

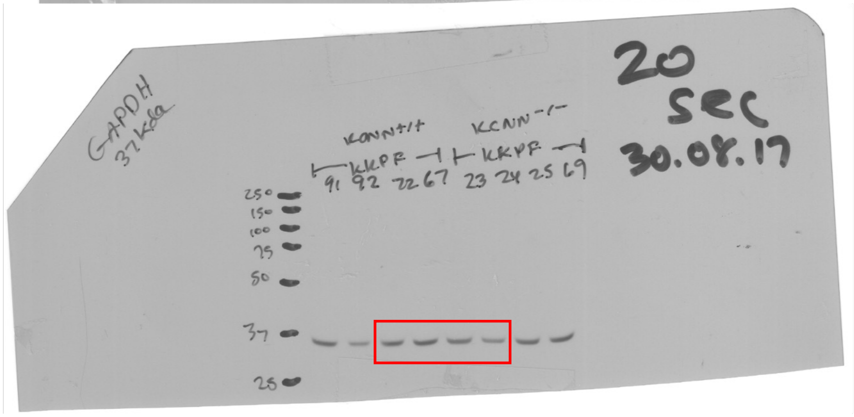

GAPDH

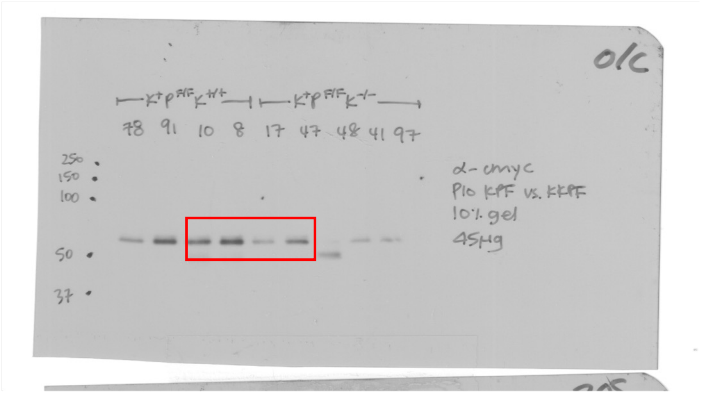

c-Myc

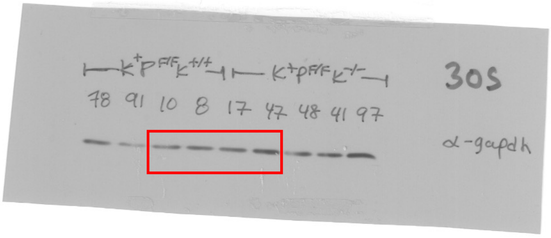

GAPDH

Figure 7K

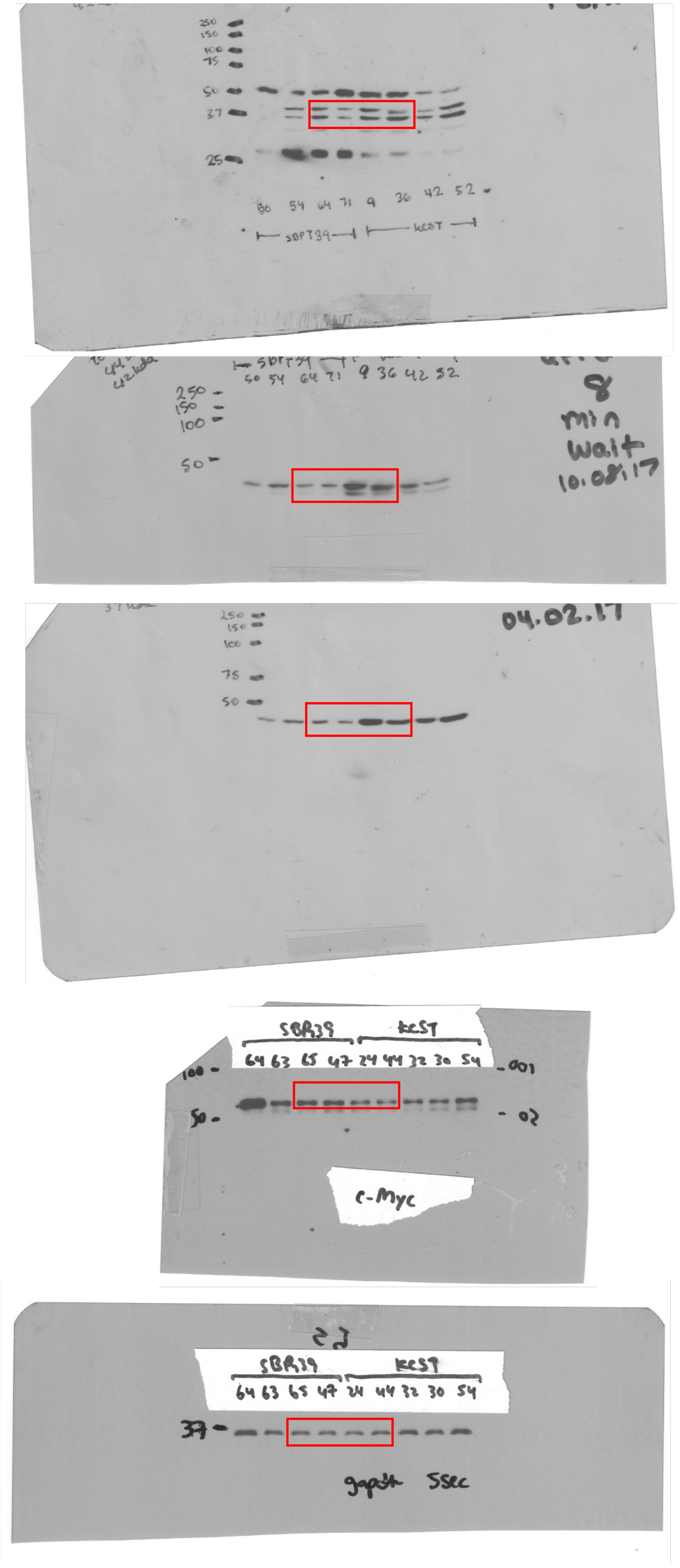

p-ERK

ERK

GAPDH

c-Myc

GAPDH

Picture S1D

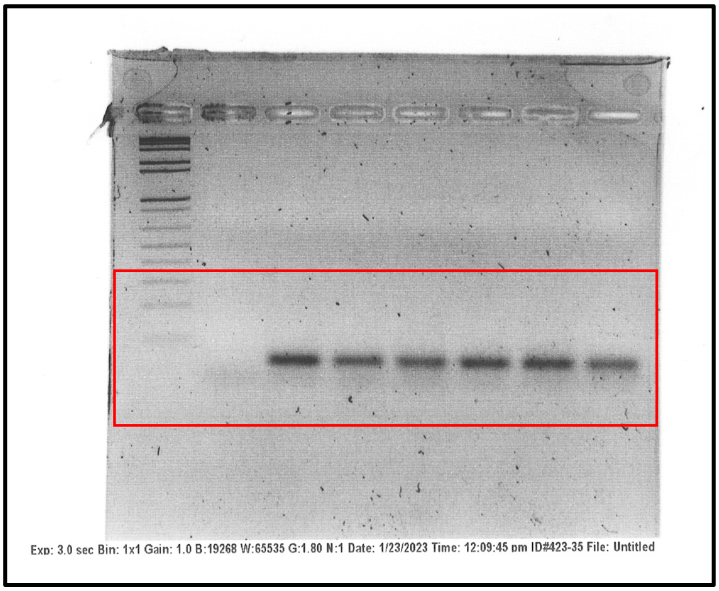

Kcnn4

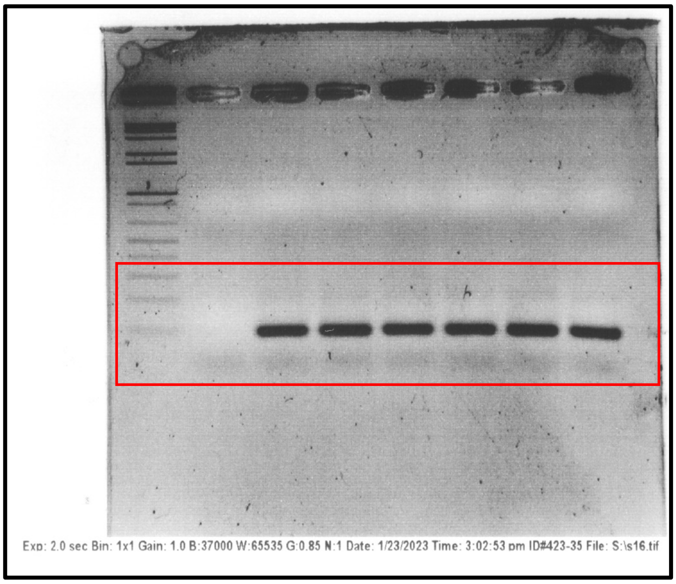

S16
